# Anti-Inflammatory and Antibacterial Activity Constituents from the Stem of *Cinnamomum validinerve*

**DOI:** 10.3390/molecules25153382

**Published:** 2020-07-25

**Authors:** Chi-Lung Yang, Ho-Cheng Wu, Tsong-Long Hwang, Chu-Hung Lin, Yin-Hua Cheng, Chia-Chi Wang, Hung-Lin Kan, Yueh-Hsiung Kuo, Ih-Sheng Chen, Hsun-Shuo Chang, Ying-Chi Lin

**Affiliations:** 1 School of Pharmacy, College of Pharmacy, Kaohsiung Medical University, Kaohsiung 807, Taiwan; long902001@yahoo.com.tw (C.-L.Y.); chuhung.lin@gmail.com (C.-H.L.); ccwang@ntu.edu.tw (C.-C.W.); m635013@kmu.edu.tw (I.-S.C.); 2 Graduate Institute of Natural Products, College of Pharmacy, Kaohsiung Medical University, Kaohsiung 807, Taiwan; duncanwu762001@gmail.com; 3 Graduate Institute of Natural Products, College of Medicine, Chang Gung University, Taoyuan 333, Taiwan; htl@mail.cgu.edu.tw; 4 Research Center for Industry of Human Ecology, Research Center for Chinese Herbal Medicine, and Graduate Institute of Health Industry Technology, College of Human Ecology, Chang Gung University of Science and Technology, Taoyuan 333, Taiwan; 5 Department of Anesthesiology, Chang Gung Memorial Hospital, Taoyuan 333, Taiwan; 6 PhD Program in Toxicology, College of Pharmacy, Kaohsiung Medical University, Kaohsiung 807, Taiwan; justjudykimo@gmail.com (Y.-H.C.); k38628511@gmail.com (H.-L.K.); 7 Department of Chinese Pharmaceutical Sciences and Chinese Medicine Resources, Chinese Medicine Research Center, and Research Center for Chinese Herbal Medicine, China Medical University, Taichung 404, Taiwan; kuoyh@mail.cmu.edu.tw; 8 Drug Development and Value Creation Research Center, Kaohsiung Medical University, Kaohsiung 807, Taiwan

**Keywords:** *Cinnamomum validinerve*, Lauraceae, anti-inflammatory activity, anti-acne activity

## Abstract

One new dibenzocycloheptene, validinol (**1**), and one butanolide firstly isolated from the natural source, validinolide (**2**), together with 17 known compounds were isolated from the stem of *Cinnamomum validinerve*. Among the isolates, lincomolide A (**3**), secosubamolide (**7**), and cinnamtannin B1 (**19**) exhibited potent inhibition on both superoxide anion generation (IC_50_ values of 2.98 ± 0.3 µM, 4.37 ± 0.38 µM, and 2.20 ± 0.3 µM, respectively) and elastase release (IC_50_ values of 3.96 ± 0.31 µM, 3.04 ± 0.23 µM, and 4.64 ± 0.71 µM, respectively) by human neutrophils. In addition, isophilippinolide A (**6**), secosubamolide (**7**), and cinnamtannin B1 (**19**) showed bacteriostatic effects against *Propionibacterium acnes* in in vitro study, with minimal inhibitory concentration (MIC) values at 16 μg/mL, 16 μg/mL, and 500 μg/mL, respectively. Further investigations using the in vivo ear *P. acnes* infection model showed that the intraperitoneal administration of the major component cinnamtannin B1 (**19**) reduced immune cell infiltration and pro-inflammatory cytokines TNF-α and IL-6 at the infection sites. The results demonstrated the potential of cinnamtannin B1 (**19**) for acne therapy. In summary, these results demonstrated the anti-inflammatory potentials of Formosan *C. validinerve* during bacterial infections.

## 1. Introduction

Lauraceous plants are distributed throughout the tropical and subtropical regions with numerous species. In Taiwan, Lauraceous plants are the main composition of forest and occupy an important position in natural resources. Besides, Lauraceous plants reveal diverse bioactivities, such as anti-tuberculosis, anti-inflammatory, cytotoxicity, and antiplatelet [1], which have garnered worldwide attention. However, still, many Lauraceous plants have never been studied before, and the purpose of this study is to explore the bioactive compounds and potential medication use from Lauraceous plants. *Cinnamomum* species is one of the famous Lauraceous plants that has economic importance, aromatic potential, and bioactive properties [2]. Especially, cinnamon extracts from *Cinnamomum* species showed potent anti-acne activity for skincare [3,4,5,6]. *Cinnamomum validinerve* Hance (*C. brevipedunculatum* C. E. Chang) is a Lauraceous plant growing in Taiwan, Hong Kong, and China [7,8], whose chemical constituents and biological activities have not been investigated before. Recently, we accomplished a series of bioactive screening of Lauraceous plants. The methanolic extract of the stem of *C. validinerve* showed anti-inflammatory and antioxidant activities, indicating its development potential. Therefore, we paid much attention to searching for additional compounds with novel structures and potent bioactivities from *C. validinerve*.

Neutrophils are famous for playing a significant role in acute inflammatory responses against infections and are also associated with chronic inflammatory and autoimmune diseases [9,10,11]. Neutrophils infiltrate to the inflamed site, generating superoxide anion and associated reactive oxygen species that exaggerate the inflammatory response further [12]. Bacterial *N*-formyl peptides formyl-l-methionyl-l-leucyl-l-phenylalanine (fMLP) are involved in the innate immunity mechanism of the host defense against pathogens and shown to be chemoattractants for neutrophils. Neutrophils release superoxide anion and elastase in response to fMLP stimulation to initiate the inflammation activity [12].

Acne vulgaris is one of the top three most prevalent skin conditions, which affects 85% of young adults aged 12–25 years, according to the Global Burden of Disease (GBD) study [13]. It is a chronic inflammatory disease, and the pathogenesis is multifactorial with four primary factors: excess sebum production, abnormal keratinization, inflammation, and bacterial colonization of *Propionibacterium acnes* in the pilosebaceous unit [14,15]. Although not fatal, persistent acne lesions or inflammation on the face could result in serious psychosocial stress to the patients [16]. Topical benzoyl peroxide (BPO) is an established treatment for acne, which helps reduce the chronic use of antibiotics and associated drug-resistant problems. However, BPO may adversely cause skin reactions such as local irritation, reddish skin, or hair bleaching at the start of treatment [17]. Intralesional corticosteroid injection is another option for acne, but it may cause some side effects such as the pitting and thinning of the skin. Thus, more treatment options for possessing anti-acne activity are needed.

**Figure 1:**
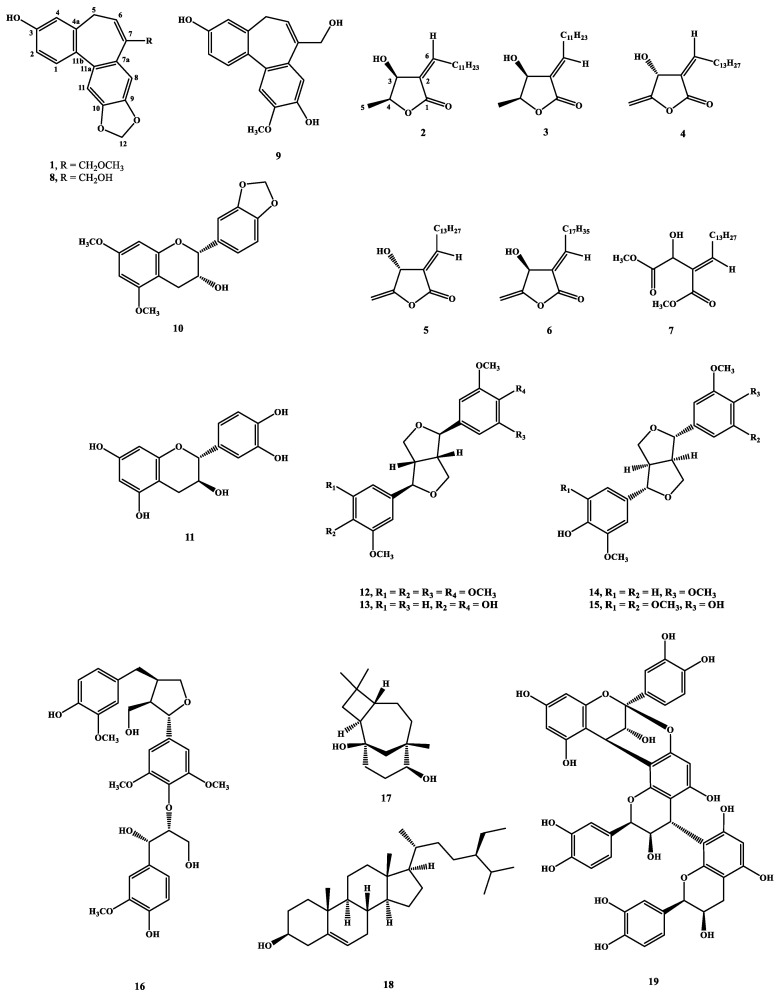
Structures of compounds **1**–**19**.

Herein, we report the structure elucidation of a new dibenzocycloheptene, validinol (**1**), a butanolide first isolated from the natural source, and validinolide (**2**), together with 17 known compounds (Figure 1) from the stem of *C. validinerve*. The phytochemical spectra of compounds **1** and **2** are available in the Supplementary Materials. Some compounds were evaluated for anti-inflammatory activity and anti-acne activity in vitro, and the major component, cinnamtannin B1 (**19**), was further evaluated as the acne treatment in vivo.

## 2. Results

**Figure 2:**
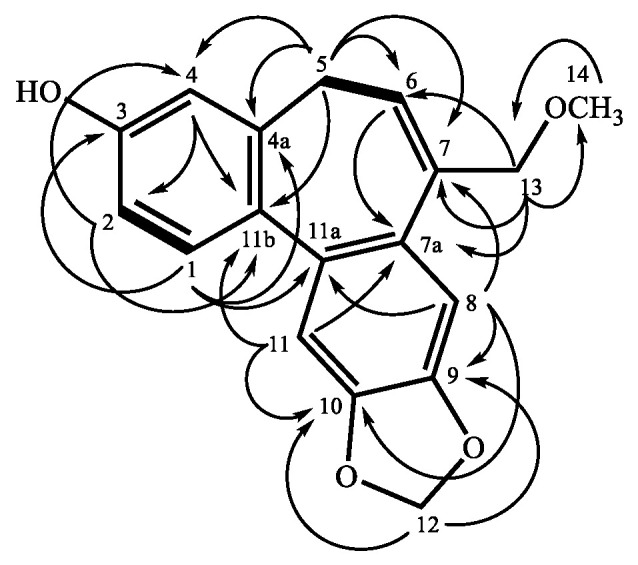
COSY (▬) and HMBC (→) correlations of **1**.

Validinol (**1**) was obtained as a pale yellowish oil. The molecular formula was established as C_18_H_16_O_4_ by HRESIMS at *m/z* 319.09420 [M + Na]^+^ (calculated for C_18_H_16_O_4_Na, 319.09408) with 11 degrees of unsaturation. The UV spectrum showed maximum absorption at 245 and 284 nm, and it showed a bathochromic shift after the addition of KOH solution, suggesting the presence of a phenolic moiety. The UV spectrum of **1** depicted typical 5*H*-dibenzo[*a*,*c*]cycloheptene derivatives [18]. The IR absorption represents the existence of hydroxyl (3301 cm^−1^), aromatic ring (1604, 1501 cm^−1^), and methylenedioxy (1035, 927 cm^−1^), respectively. The ^1^H NMR spectrum of **1** showed an *ABX* aromatic ring at *δ* 6.68 (1H, d, *J* = 2.8 Hz, H-4), 6.72 (1H, dd, *J* = 8.2, 2.8 Hz, H-2), and 7.32 (1H, d, *J* = 8.2 Hz, H-1), two singlet aromatic signals at *δ* 7.05 (1H, s, H-11) and 7.16 (1H, s, H-8), one olefinic proton at *δ* 6.15 (1H, dd, *J =* 7.9, 7.1 Hz, H-6), one methylene at *δ* 2.75 (1H, dd, *J* = 12.8, 7.1 Hz, H-5b) and 3.04 (1H, dd, *J* = 12.8, 7.9 Hz, H-5a), one oxygenated methylene at *δ* 4.04 (1H, d, *J =* 11.6 Hz, H-13b) and 4.28 (1H, br d, *J* = 11.6 Hz, H-13a), one methoxy signal at *δ* 3.19 (3H, s, H-14), one hydroxyl group at 4.97 (1H, br s, OH-3), and one methylenedioxy proton at *δ* 6.01 (1H, d, *J* = 1.2 Hz, H-12b) and 6.02 (1H, d, *J* = 1.2 Hz, H-12a). The ^13^C NMR and DEPT spectra of **1** showed 18 resonances comprising one methyl (C-14), three methylenes (C-5, 12, 13), six methines (C-1, 2, 4, 6, 8, 11), and eight quaternary carbons (C-3, 4a, 7, 7a, 9, 10, 11a, 11b). The coupling pattern between H-5 and H-6 and the COSY correlation (Figure 2) represents the existence of a prop-1-ene fragment, which was located at cycloheptene due to the HMBC correlation from H-5 to C-4, as well as C-4a, C-6, C-7, C-11b, and H-6 to C-7a. The methylenedioxy was attached to C-9 and C-10 based on the HMBC correlation between H-12 to C-9 and C-10. The HMBC correlations (Figure 2) were between OCH_3_-14 to C-13 and H-13 to C-6, C-7, and C-7a, suggesting that the CH_2_OCH_3_ fragment was bearing on C-7. Finally, the remaining OH is located at the *ABX* system aromatic ring on C-3. Thus, the entire structure of **1** was confirmed and named validinol.

Validinolide (**2**) was isolated as a colorless oil with a negative specific rotation [α]_D_^22^ −22 (*c* 0.09, CHCl_3_), and displayed a pseudo-molecular ion at *m/z* 305.20882 [M + Na]^+^ (calcd. for C_17_H_30_O_3_Na, 305.20872) by HRESIMS. The UV spectrum exhibited bands at 217 and 257 nm, indicating the presence of a butyrolactone moiety [19,20]. The IR spectrum showed the absorptions at 3387 cm^−1^ (hydroxyl group), 1727 cm^−1^, and 1692 cm^−1^ (*α*,*β*-unsaturated-*γ*-lactone). In the ^1^H NMR spectrum, **2** had the trisubstituted double bond with the *Z*-form geometry [*δ* 6.56 (1H, td, *J* = 7.6, 1.2 Hz, H-6)]. According to the above data, **2** showed the same planer structure of litsenolide C2 [20] except for the configuration. The laevorotatory optical activity suggested that the configuration of **2** could be (3*S*,4*S*) or (3*S*,4*R*) [19,21,22,23]. Besides, the chemical shifts and coupling patterns of H-3 [*δ* 4.67 (1H, br d, *J* = 4.9 Hz)] and H-4 [*δ* 4.55 (1H, qd, *J* = 6.3, 4.9 Hz, H-4)] in **2** indicated that H-3 and H-4 are *cis*-oriented, which is similar to isodihydromahubynolide B [20]. As a consequence, the absolute configurations of C-3 and C-4 were deduced to be 3*S*,4*S*. Based on this information, the structure of **2** was proposed to be (2*Z*,3*S*,4*S*)-2-(dodecylidene)-3-hydroxy-4-methylbutanolide and named validinolide, which was first isolated from nature. It has been previously synthesized and yielded as a mixture [19,20].

The known compounds lincomolide A (**3**) [24], linderanolide B (**4**) [25], isolinderanolide B (**5**) [25], isophilippinolide A (**6**) [26], secosubamolide (**7**) [27], reticuol (**8**) [28], burmanol (**9**) [29], (−)-5,7-dimethoxy-3′,4′-methylenedioxy-flavan-3-ol (**10**) [30], taxifolin (**11**) [31], (−)-yangambin (**12**) [32], (−)-pinoresinol (**13**) [33], (+)-monomethylpinoresinol (**14**) [34], (+)-syringaresinol (**15**) [33], *erythro*-guaiacylglycerol-*β*-*O*-4′-(5′)-methoxylariciresinol (**16**) [35], caryolane-1,9*β*-diol (**17**) [36], *β*-sitosterol (**18**) [37], and cinnamtannin B1 (**19**) [38] were identified by comparisons of physical and spectroscopic data ([α]_D_, UV, IR, ^1^H NMR, and MS) with authentic samples or literature data. Among them, cinnamtannin B1 (**19**) (3.6 g) was obtained as the major constituent.

In this study, the effects of isolates on neutrophil pro-inflammatory responses were evaluated by the suppression of fMLP/CB (cytochalasin B)-induced superoxide anion (O^2−^) generation and elastase release. The inhibitory activity of compounds **1**, **3**, **5**–**8**, **11**, **13**, **15**, **18**, and **19** are shown in Table 1. Compounds **3**, **7**, and **19** exhibited inhibitory activities on both superoxide anion (IC_50_ values of 2.98 ± 0.3 µM, 4.37 ± 0.38 µM, and 2.20 ± 0.3 µM) and elastase release (IC_50_ values of 3.96 ± 0.31 µM, 3.04 ± 0.23 µM, and 4.64 ± 0.71 µM), and compound **13** only showed inhibitory activity on superoxide anion with an IC_50_ value of 5.99 ± 1.77 µM.

Compounds **6**, **7**, and **19** were evaluated for their ability to inhibit the growth of *Propionibacterium acnes*. Compounds **6** and **7** showed anti-microbial activity against *P. acnes*, with a minimal inhibitory concentration (MIC) value of 16 μg/mL. Compound **19** only exhibited moderate anti-microbial activity against *P. acnes* with an MIC value of 500 μg/mL. The MIC of benzoyl peroxide, the active component of epicutaneous medications for acne treatment, as a positive control in our experiment was 1000 μg/mL.

**Figure 3:**
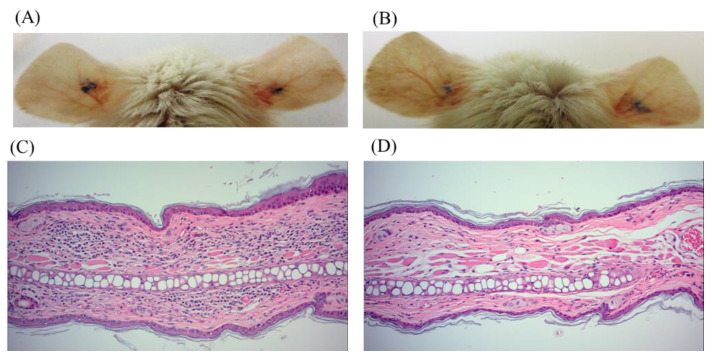
In vivo anti-inflammatory activity of cinnamtannin B1 (**19**). *P. acnes* was intradermally injected into the ears at the blue-marked positions, and phosphate-buffered solution (PBS) or **19** (20 mg/kg) were administered intraperitoneally daily for 4 days starting right after the infection. Representative photos of mice (**A**) PBS-treated and (**B**) **19**-treated 24 h after the infection. (**C**) A PBS-treated histology section (H&E staining) (**D**) a **19**-treated histology section (H&E staining) on day 5 after the infection. The sections were 200×-magnified.

**Figure 4:**
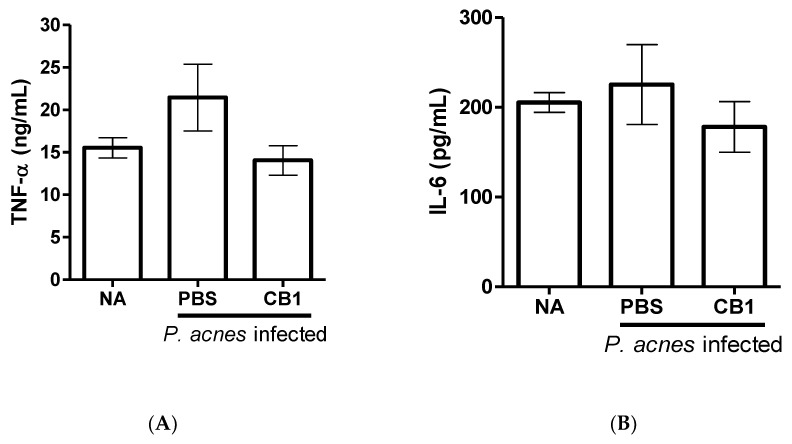
Anti-inflammatory effect of compound **19** in *P. acnes* ear infection model. (**A**) TNF-α, (**B**) IL-6. The data were pooled from two independent experiments. Error bar indicated the standard deviation of the mean (SEM). Control: naïve mice (N = 2); PBS: *P. acnes* infected, PBS-treated mice (N = 6); CB1: *P. acnes* infected, **19** (20 mg/kg)-treated mice (N = 7). *p*-value between PBS and CB1 for TNF-α and IL-6 were 0.098 and 0.377, respectively.

As cinnamtannin B1 (**19**) is the major component in the stem of *C. validinerve* and showed anti-inflammatory and anti-*P. acnes* activities in vitro, we further examined the potential of cinnamtannin B1 (**19**) as an acne treatment with an in vivo *P. acnes* ear infection model. At the dosage of 20 mg/kg administered intraperitoneally, cinnamtannin B1 (**19**) was able to reduce the redness of the ears starting from 24 h (Figure 3A,B). Furthermore, cinnamtannin B1 (19) decreased immune cell infiltration in the mouse ears infected with *P. acnes* (Figure 3C,D) and trended to decrease the inflammatory cytokines TNF-α (*p* = 0.098) and IL-6 levels (*p* = 0.377) associated with the infection (Figure 4). No significant decrease in bacterial load was observed (1.08 × 10^6^ CFU/mL versus 3.10 × 10^6^ CFU/mL in treated versus control ears).

## 3. Discussion

Inflammation is a part of the innate immune mechanism to defense infection or tissue injury. However, prolonged, dysregulated, or excessive inflammation could adversely cause tissue destruction and human diseases. Traditionally, *Cinnamomum* is used for flavoring food and pharmaceutical medications around the world. Although some studies reported the anti-inflammatory activity from *Cinnamomum,* this is the first time evaluating the inhibition effects of *Cinnamomum* compounds on superoxide anion and elastase release in fMLP/CB-activated human neutrophils. Focusing on the anti-inflammatory activity results in this paper, compounds **3**, **7**, and **19** exhibited inhibitory activities on superoxide anion and elastase release. The results in chemistry also contributed to the chemotaxonomy of *Cinnamomum* species. According to the in vitro anti-microbial activities against *P. acnes*, cinnamtannin B1 (**19**) was better than BPO in its antibacterial activity against *P. acnes*. Cinnamtannin B1 (**19**) also showed good anti-inflammatory activity in vivo, which was shown to relieve the inflammation associated with *P. acne* infections. By contrast, BPO was too irritating to be administered systematically. Therefore, cinnamtannin B1 (**19**) has the potential to be further evaluated for anti-acne therapy in humans, especially if topical formulations can be developed. The combination of cinnamtannin B1 (**19**) with other currently available treatments for acne may also be worthy of further evaluation to decrease the use of antibiotics in the treatment of the chronic condition and/or to potentiate anti-acne treatment effects for other agents with different mechanisms of action [39].

Three compounds, namely isophilippinolide A (**6**), secosubamolide (**7**), and cinnamtannin B1 (**19**), were reported with anti-*P. acnes* activities. Although the tannin compound “cinnamtannin B1 (**19**)” was not the compound with the best anti-inflammatory activity nor anti-*P. acnes* activity, the abundance of the compound in this plant enables us to perform a pilot study *P. acnes* infection experiment in vivo. It is worth mentioning that a reduction in the *P. acnes*-induced redness can be observed from 24 h after the infection, and reduced immune cell infiltration and pro-inflammation cytokines, TNF-α and IL-6, were maintained and observed on day 5. The mouse-ear infection model was chosen because its confined region can preserve all inoculated bacteria in the injection area. However, given that cinnamtannin B1 (**19**) was administered intraperitoneally, the concentration of cinnamtannin B1 (**19**) at the ears may be too low to reduce the bacterial load at the infection sites. The reduction in the signs and symptoms of inflammation was likely due to the anti-inflammatory activity of cinnamtannin B1 (**19**). The observations implied that cinnamtannin B1 may achieve better anti-acne property if the dose or administration method can be further optimized.

## 4. Conclusions

One new dibenzocycloheptene, validinol (**1**), and one butanolide firstly isolated from the natural source, validinolide (**2**), together with 17 known compounds, were obtained from the stem of *C. validinerve.* Six butanolides are successfully isolated from *C. validinerve*, which were the most major skeleton in this study. These phytochemical results fitted with the previous investigation that butanolides are abundant in *Cinnamomum* genus [1]. This result also contributed to the chemotaxonomy of *Cinnamomum* genus. For the treatment of acne vulgaris, we targeted two main pathogenesis factors, inflammation and *P. acne,* for the treatment of acne vulgaris. This report also demonstrated that *C. validinerve* contains some butanolide and tannin compounds with anti-inflammatory activity and anti-acne activity, which is helpful to patients with inflammation-related disease.

## 5. Materials and Methods

### 5.1. General Experimental Procedures

All melting points were determined on a Yanaco micromelting apparatus (Yanaco, Kyoto, Japan) and were uncorrected. Optical rotations were measured on a Jasco P-2000 polarimeter (Jasco, Kyoto, Japan), UV spectra were obtained with a Jasco-V-530 UV/vis spectrophotometer (Jasco, Kyoto, Japan), and IR spectra (ATR) were acquired with a Jasco FT/IR-4600 spectrometer. 1D (^1^H, ^13^C, DEPT) and 2D (COSY, NOESY, ROESY, HSQC, HMBC) NMR spectra were recorded on a Varian Germini-2000 spectrometer (Varian, Inc. Vacuum Technologies, MA, USA) operated at 200 MHz (^1^H) and 50 MHz (^13^C), a Varian Unityplus-400 spectrometer (Varian, Inc. Vacuum Technologies, MA, USA) operated at 400 MHz (^1^H) and 100 MHz (^13^C), a Varian Mercuryplus-400 spectrometer (Varian, Inc. Vacuum Technologies, MA, USA) operated at 400 MHz (^1^H) and 100 MHz (^13^C), and a Varian VNMRS-600 spectrometer (Varian, Inc. Vacuum Technologies, Lexington, MA, USA) operated at 600 MHz (^1^H) and 150 MHz (^13^C). Low-resolution mass spectra were obtained with POLARIS Q Thermo Finnigan (Thermo Fisher Scientific, Chicago, IL, USA), Water ZQ 4000 (Waters, Milford, MA, USA), and VG Quattro GC/MS/MS/DS (Waters, Milford, MA, USA) mass spectrometers. HRESIMS were recorded on a Bruker APEX II mass spectrometer (Bruker, Karlsruhe, Germany). Silica gel (70–230 and 230–400 mesh; Silicycle, Quebec, Canada) was used for column chromatography (CC), and silica gel 60 F254 (Merck, Darmstadt, Germany) and RP-18 F254S (Merck, Darmstadt, Germany) were used for thin-layer chromatography (TLC) and preparative TLC, respectively, which were visualized with Ce_2_(SO4)_3_ aqueous solution. Further purification was performed by medium-performance liquid chromatography (MPLC; ceramic pump: VSP-3050; EYELA, Kyoto, Japan).

### 5.2. Plant Material

The stem of *C. validinerve* was collected from Mudan, Pingtung County, Taiwan, in January 2013 and identified by I.-S. C. A voucher specimen (Chen 2321) was deposited in the School of Pharmacy, Kaohsiung Medical University, Kaohsiung, Taiwan.

### 5.3. Extraction and Isolation

The dried stem (6.5 kg) were extracted with MeOH, obtaining a MeOH extract (350 g). The MeOH extract was partitioned into an ethyl acetate-soluble fraction (200 g) and H_2_O-soluble fraction (120 g). The active ethyl acetate-soluble fraction (100 g) was chromatographed over silica gel using an *n*-hexane–acetone gradient to yield 15 fractions (Fraction 1–Fraction 15). Fraction 2 (9.2 g) was chromatographed by silica gel eluting with *n-*hexane–acetone gradient to give 37 fractions. Fraction 2-20 was purified via a silica gel column using *n*-hexane-ethyl acetate (10:1) to afford **6** (12.6 mg) and **7** (9.8 mg). Fraction 3 was submitted to silica gel column eluting with *n-*hexane–ethyl acetate (10:1) to give 17 fractions and to obtain **5** (44.8 mg). Fraction 3-14 was chromatographed by silica gel eluting with dichloromethane-ethyl acetate (50:1) to give 10 fractions. Fraction 3-14-7 was eluted with H_2_O–methanol (1:10) through RP-18 column to afford **18** (15.4 mg). Fraction 4 was submitted to silica gel column eluting with *n*-hexane-ethyl acetate (7:1) to give 18 fractions. Fraction 4-14 was purified by preparative RP-18 TLC (H_2_O–methanol, 1:5) to give **2** (2.5 mg) and **3** (2.7 mg). Fraction 4-4 was purified by preparative RP-18 TLC (H_2_O–acetone, 1:5) to furnish **4** (2.0 mg). Fraction 5 was subjected to a silica gel column with *n*-hexane–acetone (4:1) and was purified by preparative TLC (dichloromethane–acetone, 30:1) to produce **1** (2.3 mg) and **10** (1.3 mg). Fraction 7 was eluted with dichloromethane–methanol (30:1) by silica gel column to gain 14 fractions. Fraction 7-2 was purified on RP-18 silica gel eluting with H_2_O-methanol (1:2) to generate **12** (0.6 mg). Both Fraction 7-3 and Fraction 7-6 were purified via an RP-18 column eluting with H_2_O–methanol (1:2) to give **14** (0.4 mg) and **9** (0.7 mg). Fraction 7-12 was purified by an RP-18 column eluting with H_2_O–methanol (1:2) to obtain **17** (1.8 mg). Fraction 8 was chromatographed by RP-18 silica gel eluting with H_2_O–methanol (1:1) to give 11 fractions. Both Fraction 8-2 and Fraction 8-4 were purified by preparative TLC (dichloromethane–methanol, 30:1) to produce **13** (7.0 mg) and **8** (2.4 mg). Fraction 9 was submitted to a silica gel column eluting with dichloromethane–methanol (15:1) to give nine fractions. Fraction 9-5 was purified via silica gel eluting with *n*-hexane–acetone (3:2) to give nine fractions. Fraction 9-5-7 was purified by preparative RP-18 TLC (H_2_O–methanol, 1:1) to provide **11** (24.3 mg). Fraction 9-3 was purified by RP-18 column eluting with H_2_O–acetone (3:2) to obtain **15** (5.4 mg). Fraction 11 went through an RP-18 column eluting with dichloromethane–methanol (20:1) and provided seven fractions. Fraction 11-3 was isolated via an RP-18 column eluting with H_2_O–acetonitrile (2:1) and was purified by preparative TLC (*n*-hexane–acetone–methanol, 1:1:0.1) to furnish **16** (1.9 mg). Fraction 12 was subjected to silica gel column eluting with dichloromethane–methanol (5:1), gradually increasing the polarity with methanol to obtain **19** (3.6 g).

### 5.4. Experimental Data of Isolates

*Validinol* (**1**): pale yellowish oil; UV λ_max_ (MeOH) (log ε): 245 (4.76), 284 (4.59) nm; UV λ_max_ (MeOH + KOH) (log ε): 252 (4.79), 298 (4.72) nm; IR *v*_max_ (ATR): 3301 (OH), 1604, 1501 (aromatic ring), 1035, 927 (OCH_2_O) cm^−1^; ESIMS *m/z*: 319 [M + Na]^+^; HRESIMS *m/z*: 319.09420 [M + Na]^+^ (calcd. for C_18_H_16_O_4_Na, 319.09408). ^1^H NMR (CDCl_3_, 400 MHz) *δ*: 2.75 (1H, dd, *J* = 12.8, 7.1, H-5b), 3.04 (1H, dd, *J* = 12.8, 7.9, H-5a), 3.19 (3H, s, H-14), 4.04 (1H, d, *J* = 11.6, H-13b), 4.28 (1H, d, *J* = 11.6, H-13a), 4.97 (1H, br s, OH-3, D_2_O exchangeable), 6.01 (1H, d, *J* = 1.2, H-12b), 6.02 (1H, d, *J* = 1.2, H-12a), 6.15 (1H, dd, *J* = 7.9, 7.1, H-6), 6.68 (1H, d, *J* = 2.8, H-4), 6.72 (1H, dd, *J* = 8.2, 2.8, H-2), 7.05 (1H, s, H-11), 7.16 (1H, s, H-8), 7.32 (1H, d, *J* = 8.2, H-1). ^13^C NMR (CDCl_3_, 100 MHz) *δ*: 33.0 (C-5), 57.4 (C-14), 75.9 (C-13), 101.2 (C-12), 106.3 (C-8), 109.4 (C-11), 112.8 (C-4), 113.1 (C-2), 129.8 (C-6), 130.4 (C-7a), 130.7 (C-1), 131.4 (C-11b), 134.30 (C-11a), 134.35 (C-7), 143.7 (C-4a), 146.3 (C-9 and C-10), 155.2 (C-3).

*Validinolide* (**2**): colorless oil; [α]_D_^22^ –22.0 (*c* 0.09; CHCl_3_); UV λ_max_ (MeOH) (log *ε*): 217 (3.73), 257 (2.92) nm; IR *v*_max_ (ATR): 3387 (OH), 1727, 1692 (*α,β*-unsaturated-*γ*-lactone) cm^−1^; ESIMS *m*/*z*: 283 [M + H]^+^. HRESIMS *m*/*z*: 305.20882 [M + Na]^+^ (calcd. for C_17_H_30_O_3_Na, 305.20872); ^1^H NMR (CDCl_3_, 200 MHz) *δ* 0.88 (3H, t, *J* = 6.4 Hz, H-17), 1.26 (16H, br s, H-9–H-16), 1.40 (3H, d, *J* = 6.3 Hz, H-5), 1.59 (2H, m, H-8), 2.73 (2H, m, H-7), 4.55 (1H, qd, *J* = 6.3, 4.9 Hz, H-4), 4.67 (1H, br d, *J* = 4.9 Hz, H-3), 6.56 (1H, td, *J* = 7.6, 1.2 Hz, H-6).

### 5.5. Superoxide Anion and Elastase Release Assays

The ability of testing compounds to modulate superoxide anion generation and elastase release by neutrophils was evaluated as those of the references published by co-author Professor Tsong-Long Hwang [40,41]. The superoxide generation assay was based on the reduction of ferricytochrome c by superoxide dismutase (SOD). The elastase release assay was performed by measuring the changes in elastase substrate MeO-Suc-Ala-Ala-Pro-Val-*p*-nitroanilide. Human neutrophils (6 × 10^5^ cells/mL) were pre-incubated with ferricytochrome *c* (0.5 mg/mL) at 37 °C, and then testing compounds were added for 5 min before activation by fMLF (0.1 μM) in the presence of cytochalasin B (1 μg/mL). The absorbance at 550 nm was monitored continuously using a spectrophotometer. Human neutrophils (6 × 10^5^ cells/mL) were pre-incubated with elastase substrate (methoxysuccinyl-Ala-Ala-Pro-Val-p-nitroanilide, 100 μM) at 37 °C, and then testing compounds were added for 5 min before activation by fMLF (0.1 μM) in the presence of cytochalasin B (0.5 μg/mL). The absorbance at 405 nm was monitored continuously using a spectrophotometer. The results are expressed as a percentage of the rate of elastase release in the fMLP/CB-activated under DMSO (solvent, 0.1%). PI3K inhibitor LY29002 served as positive controls for the neutrophil assays. All assays were repeated three times. Results are presented as mean ± standard error of the mean (SEM). The student’s *t*-test was used to compare the test compound with DMSO control. A probability less than 0.05 was considered significant.

### 5.6. Minimum Inhibitory Concentration (MIC)

The antibacterial activities of the test compounds were evaluated by the broth microdilution method, according to the recommendations by Clinical and Laboratory Standard Institute (CLSI) guidelines. *Propionibacterium acnes* BCRC10723 (ATCC 6919) was obtained from the Bioresource Collection and Research Center (BCRC, Hsinchu, Taiwan).

Briefly, *P acnes* was cultured on the anaerobic blood agar plate at 37 °C for 72 h under anaerobic conditions. The bacteria were washed and re-suspended to Reinforced Clostridial Medium (RCM). Test compounds were twofold serial diluted in RCM. The inoculum was approximately 5 × 10^5^ Colony forming unit (CFU)/mL. The 96-well MIC plates were incubated at 37 °C under the anaerobic condition for 72 h. The MICs were defined as the lowest concentrations of the test compounds without visible bacterial growth. All tests were performed in duplicate.

### 5.7. Propionibacterium Acnes Mice Infection Model

A mouse ear infection model was used [17]. Experiments involving animal studies were reviewed, approved, and performed under the regulatory supervision of Kaohsiung Medical University’s Institutional Animal Care and Use Committee (IACUC, no. 106015). Briefly, *P. acnes* was cultured on anaerobic blood agar plate under the anaerobic condition at 37 °C for three days. The bacteria were taken from the plate and distributed into PBS. The ears of BALB/c mice were injected with about 5 × 10^6^ CFU of *P. acnes* in 10 or 20 μL phosphate-buffered solution (PBS). The ears of mice without bacteria served as background controls (NA). The stock concentration of cinnamtannin B1 (**19**) was 20 mg/mL in PBS. Cinnamtannin B1 (**19**) (20 mg/kg) was intraperitoneally administered once daily for four days, starting immediately after the infection. In the control group, mice were injected with PBS.

The ears of the mice were collected on Day 5 for bacterial culture, cytokine measurements, and histology. The ears were weighted and homogenized in twofold volume (μL/mg) of PBS on ice. The differences in cytokine levels between cinnamtannin B1 (**19**)-treated and the control group were analyzed using an unpaired t-test. The skin sections were paraffin-embedded, cut into 3 µm-thick slides, and hematoxylin and eosin stained.

## Figures and Tables

**Table 1:** Effect of compounds on superoxide anion generation and elastase release in *N*-formyl peptides formyl-l-methionyl-l-leucyl-l-phenylalanine (fMLP)/CB-stimulated human neutrophils.

Compound	Superoxide Anion		Elastase Release	
	IC_50_ (µM) ^a^	Inhibition ^b^ (%)	IC_50_ (µM)	Inhibition (%)
validinol (**1**)	>10	17.16 ± 3.69 *	>10	17.34 ± 6.84 *
lincomolide A (**3**)	2.98 ± 0.30	93.27 ± 2.66 *	3.96 ± 0.31	66.89 ± 6.72 *
isolinderanolide B (**5**)	>10	11.58 ± 2.43 *	>10	23.16 ± 5.04 *
isophilippinolide A (**6**)	>10	4.94 ± 7.00	>10	14.36 ± 3.10 *
secosubamolide (**7**)	4.37 ± 0.38	80.25 ± 5.45 *	3.04 ± 0.23	101.07 ± 6.68 *
reticuol (**8**)	>10	−0.91 ± 0.30 *	>10	−17.28 ± 6.65 *
taxifolin (**11**)	>10	30.04 ± 4.30 *	>10	17.09 ± 2.63
(−)-pinoresinol (**13**)	5.99 ± 1.77	65.61 ± 7.51 *	>10	20.64 ± 3.41 *
(+)-syringaresinol (**15**)	>10	2.91 ± 0.92 *	>10	2.16 ± 4.41
*β*-sitosterol (**18**)	>10	11.80 ± 6.57	>10	−6.48 ± 4.17
cinnamtannin B1 (**19**)	2.20 ± 0.30	91.58 ± 4.04 *	4.64 ± 0.71	71.27 ± 5.08 *
LY294002 ^c^	2.17 ± 0.53		6.38 ± 1.72	

^a^ Concentration necessary for 50% inhibition. ^b^ Inhibition percentage examined at samples concentration of 10 μM. The data represent the mean ± SEM. (n = 3 or 4). * *p* < 0.05 compared with the control. ^c^ positive control.

## Supplementary Materials

The following are available online, including the phytochemical spectra of compounds **1** and **2**, and the experimental data of known compounds.
