# Supplementary material for: Anti-Inflammatory and Antibacterial Activity Constituents from the Stem of Cinnamomum validinerve
Source: Molecules. 2020 Jul 25;25(15):3382. doi: 10.3390/molecules25153382 (PMC7435785; doi:10.3390/molecules25153382)
Supplement: Supplementary file 1 [file molecules-25-03382-s001.docx]

Anti-Inflammatory and Antibacterial Activity Constituents from the Stem of *Cinnamomum validinerve*

Chi-Lung Yang ^1,†^, Ho-Cheng Wu ^2,†^, Tsong-Long Hwang ^3,4,5^, Chu-Hung Lin ^1^, Yin-Hua Cheng ^6^, Chia-Chi Wang ^1^, Hung-Lin Kan ^6^, Yueh-Hsiung Kuo ^7^, Ih-Sheng Chen ^1^, Hsun-Shuo Chang ^1,2,8,^* and Ying-Chi Lin ^1,6,^*

^1^ School of Pharmacy, College of Pharmacy, Kaohsiung Medical University, Kaohsiung 807, Taiwan; long902001@yahoo.com.tw (C.-L.Y.); chuhung.lin@gmail.com (C.-H.L.); ccwang@ntu.edu.tw (C.-C.W.); m635013@kmu.edu.tw (I.-S.C.)

^2^ Graduate Institute of Natural Products, College of Pharmacy, Kaohsiung Medical University, Kaohsiung 807, Taiwan; [duncanwu762001@gmail.com](mailto:duncanwu762001@gmail.com) (H.-C.W.)

^3^ Graduate Institute of Natural Products, College of Medicine, Chang Gung University, Taoyuan 333, Taiwan; [htl@mail.cgu.edu.tw](mailto:htl@mail.cgu.edu.tw) (T.-L.H.)

^4^ Research Center for Industry of Human Ecology, Research Center for Chinese Herbal Medicine, and Graduate Institute of Health Industry Technology, College of Human Ecology, Chang Gung University of Science and Technology, Taoyuan 333, Taiwan

^5^ Department of Anesthesiology, Chang Gung Memorial Hospital, Taoyuan 333, Taiwan

^6^ PhD Program in Toxicology, College of Pharmacy, Kaohsiung Medical University, Kaohsiung 807, Taiwan; justjudykimo@gmail.com (Y.-H.C.); k38628511@gmail.com (H.-L.K.)

^7^ Department of Chinese Pharmaceutical Sciences and Chinese Medicine Resources, Chinese Medicine Research Center, and Research Center for Chinese Herbal Medicine, China Medical University, Taichung 404, Taiwan; [kuoyh@mail.cmu.edu.tw](mailto:kuoyh@mail.cmu.edu.tw) (Y.-H.K.)

^8^ Drug Development and Value Creation Research Center, Kaohsiung Medical University, Kaohsiung 807, Taiwan

***** Correspondence: hschang@kmu.edu.tw (H.-S.C.); yclin@kmu.edu.tw (Y.-C.L.); Tel.: +886-7-312-1101 (ext. 2664) (H.-S.C.); +886-7-312-1101 (ext. 2012) (Y.-C.L.)

† These authors are equally contributed to this work and should be regarded as co-first authors.

Received: 20 June 2020; Accepted: 23 July 2020; Published: date

**The List of Supplementary Material**

[Figure A1. ^1^H NMR spectrum of **1** (400 MHz in CDCl_3_) 3](#_Toc43233212)

[Figure A2. ^13^C NMR spectrum of **1** (100 MHz in CDCl_3_) 3](#_Toc43233213)

[Figure A3. DEPT spectrum of **1** 4](#_Toc43233214)

[Figure A4. COSY spectrum of **1** 4](#_Toc43233215)

[Figure A5. HSQC spectrum of **1** 5](#_Toc43233216)

[Figure A6. HMBC spectrum of **1** 5](#_Toc43233217)

[Figure A7. NOESY spectrum of **1** 6](#_Toc43233218)

[Figure A8. HRESIMS spectrum of **1** 6](#_Toc43233219)

[Figure A9. IR spectrum of **1** 7](#_Toc43233220)

[Figure A10. ^1^H NMR spectrum of **2** (600 MHz in CDCl_3_) 7](#_Toc43233221)

[Figure A11. HRESIMS spectrum of **2** 8](#_Toc43233222)

[Figure A12. IR spectrum of **2** 8](#_Toc43233223)

Experimental data of known compounds 9


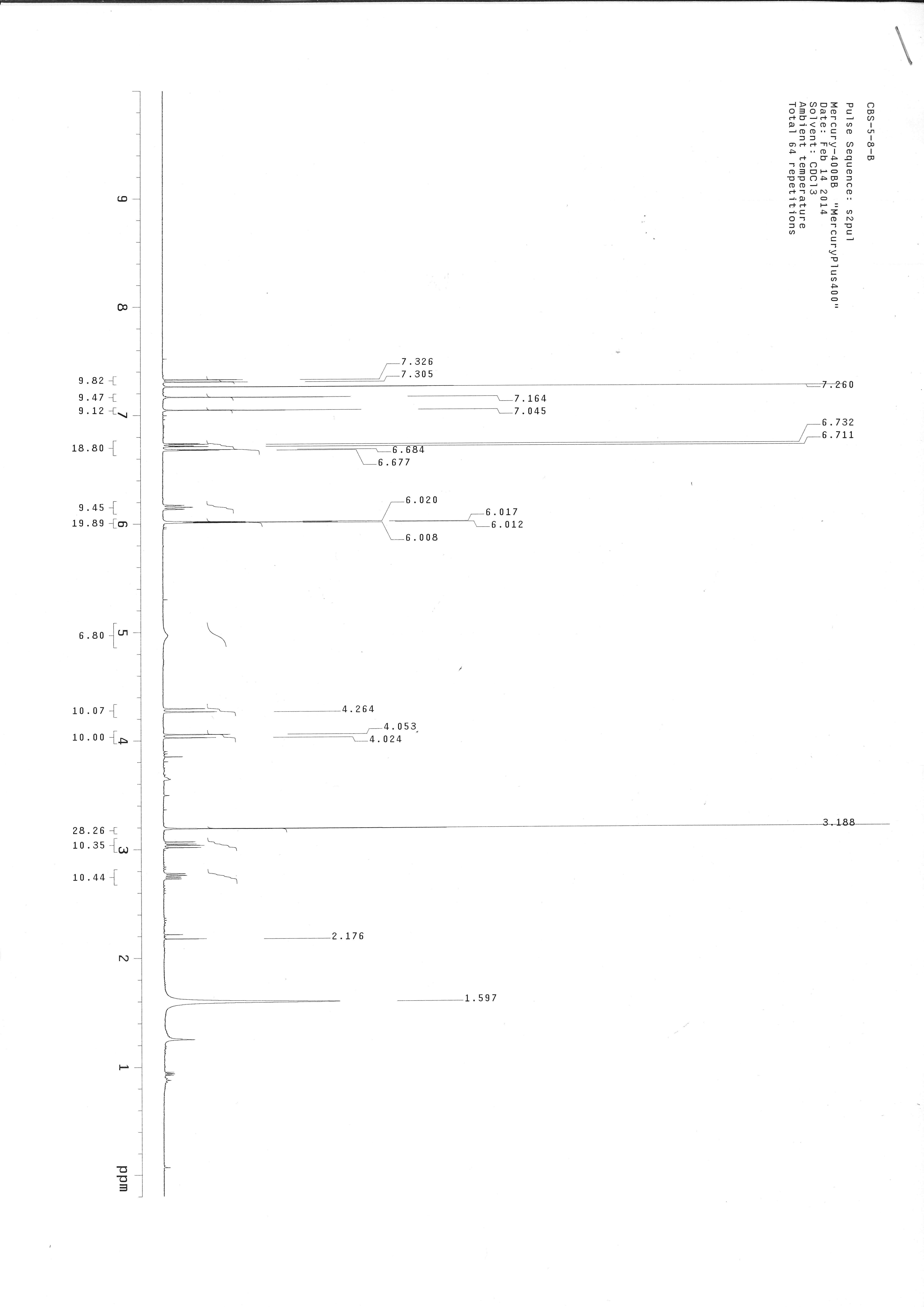


Figure A1. ^1^H NMR spectrum of **1** (400 MHz in CDCl_3_).


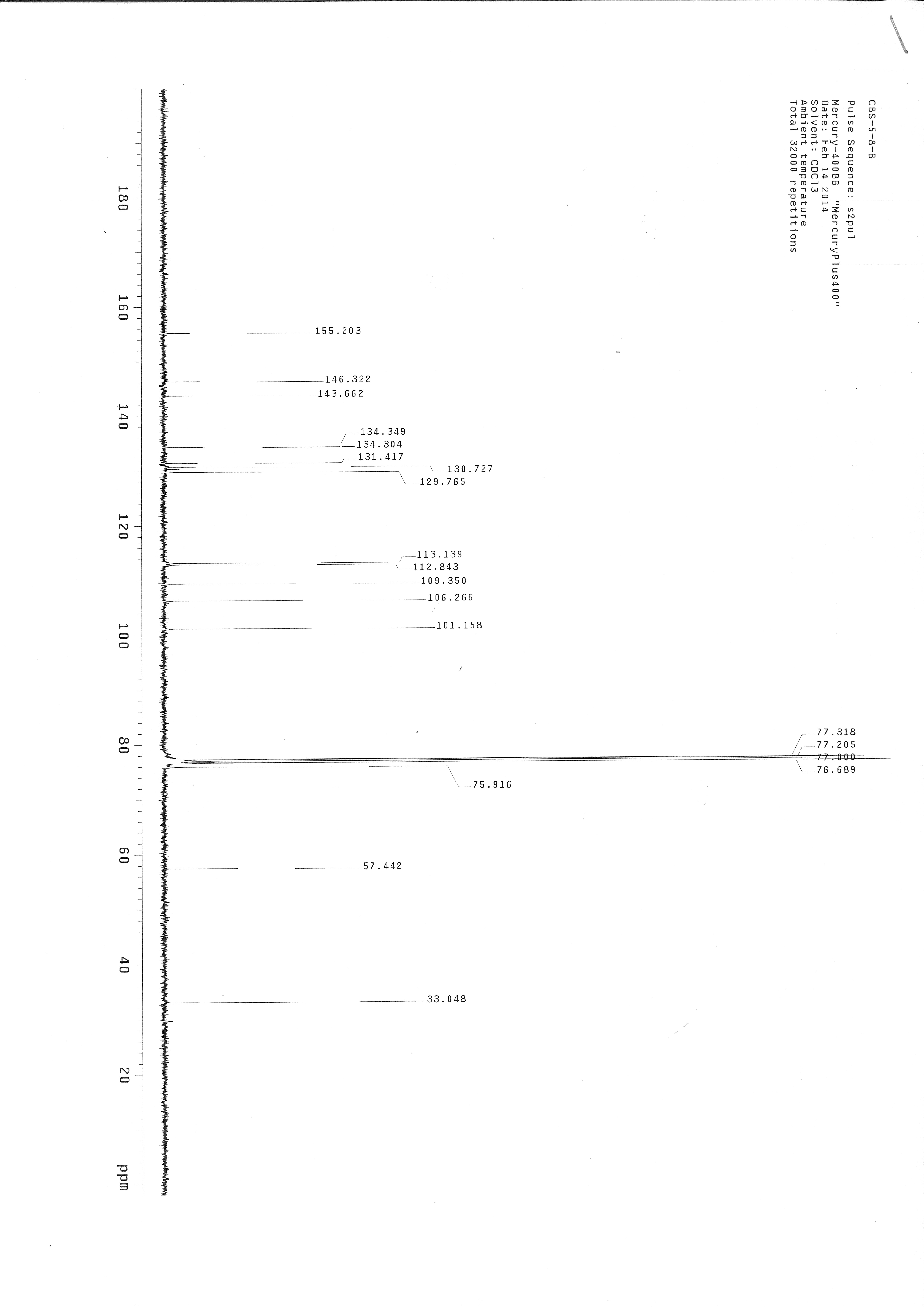


Figure A2. ^13^C NMR spectrum of **1** (100 MHz in CDCl_3_).


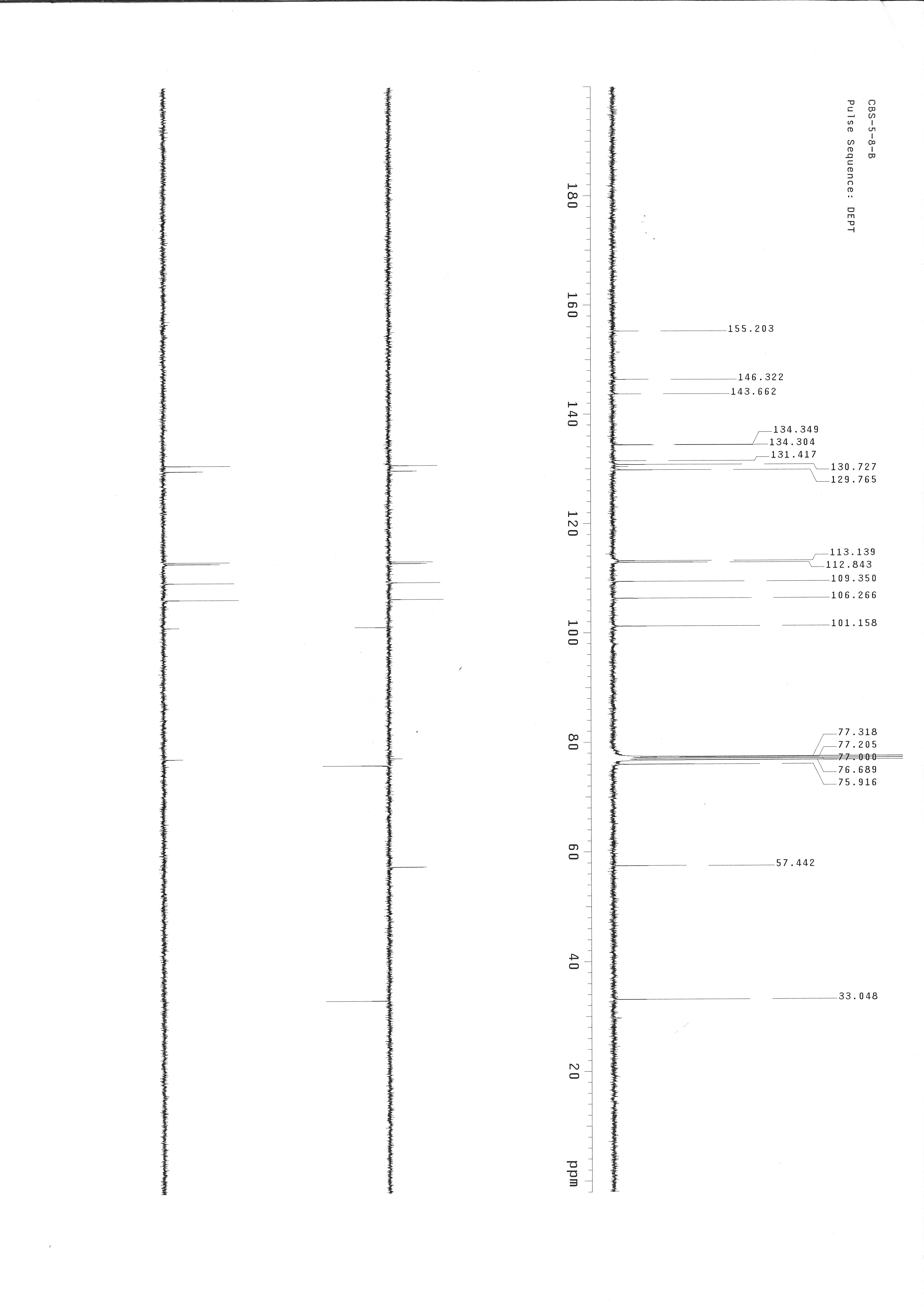


Figure A3. DEPT spectrum of **1.**


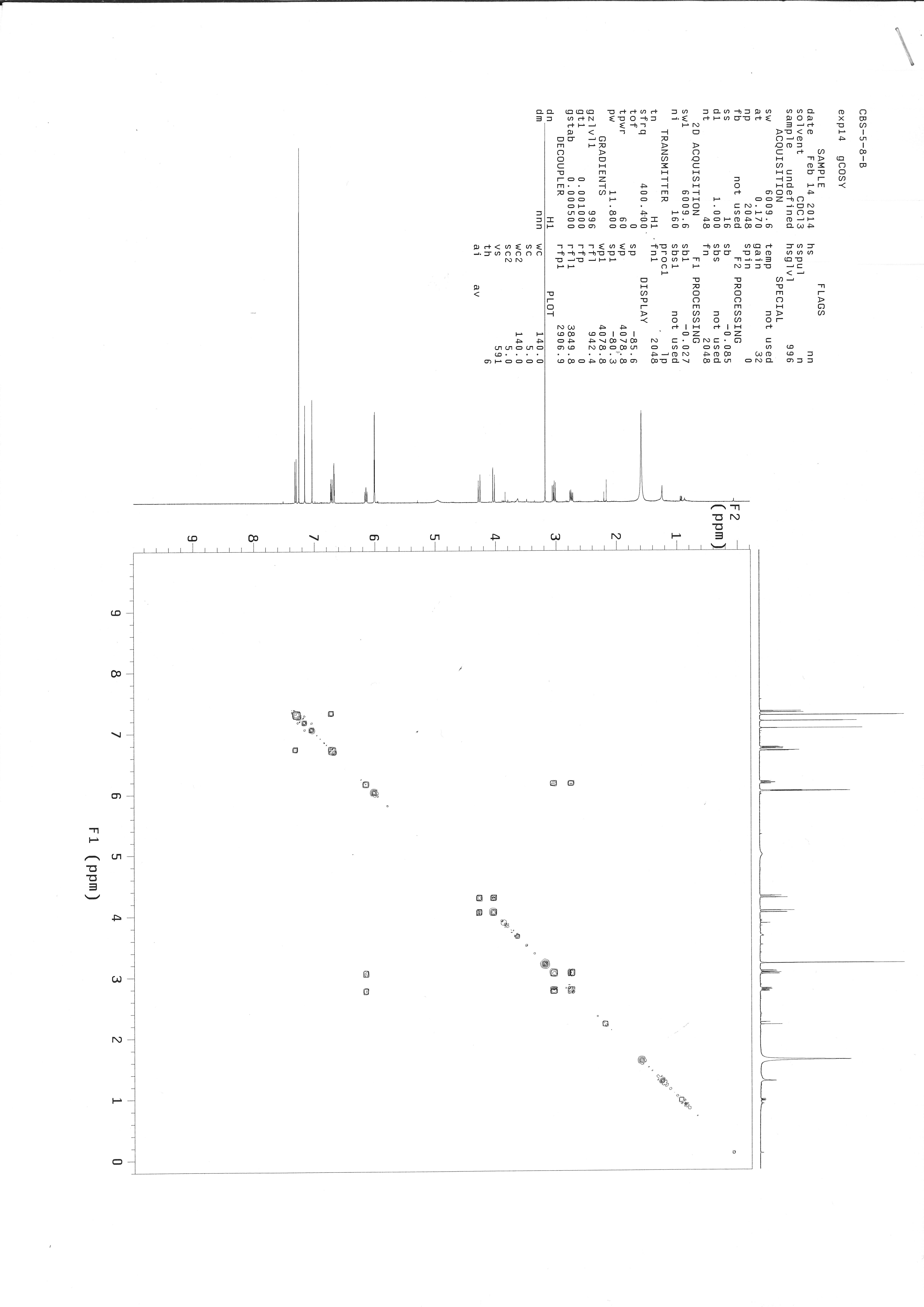


Figure A4. COSY spectrum of **1.**


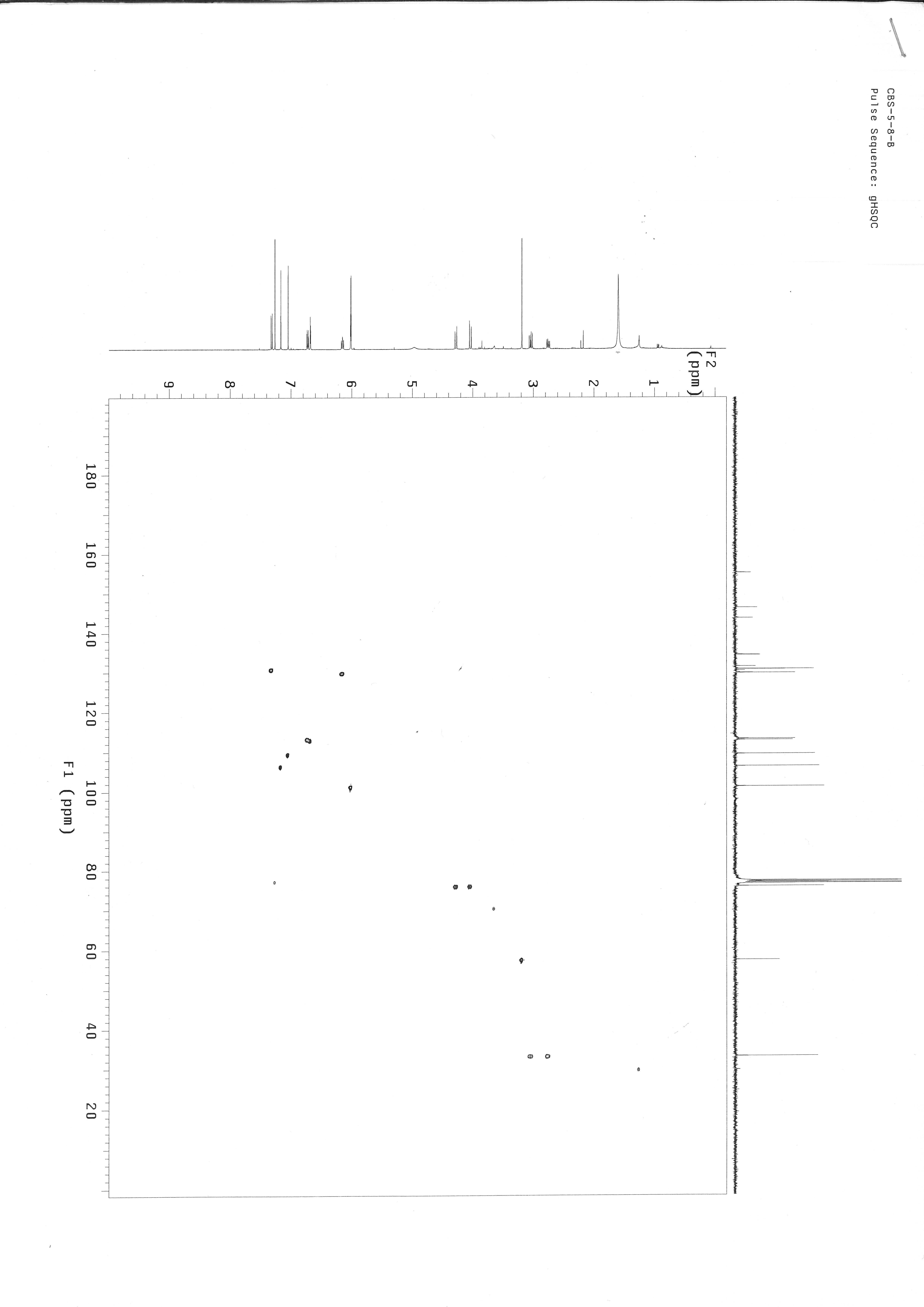


Figure A5. HSQC spectrum of **1.**


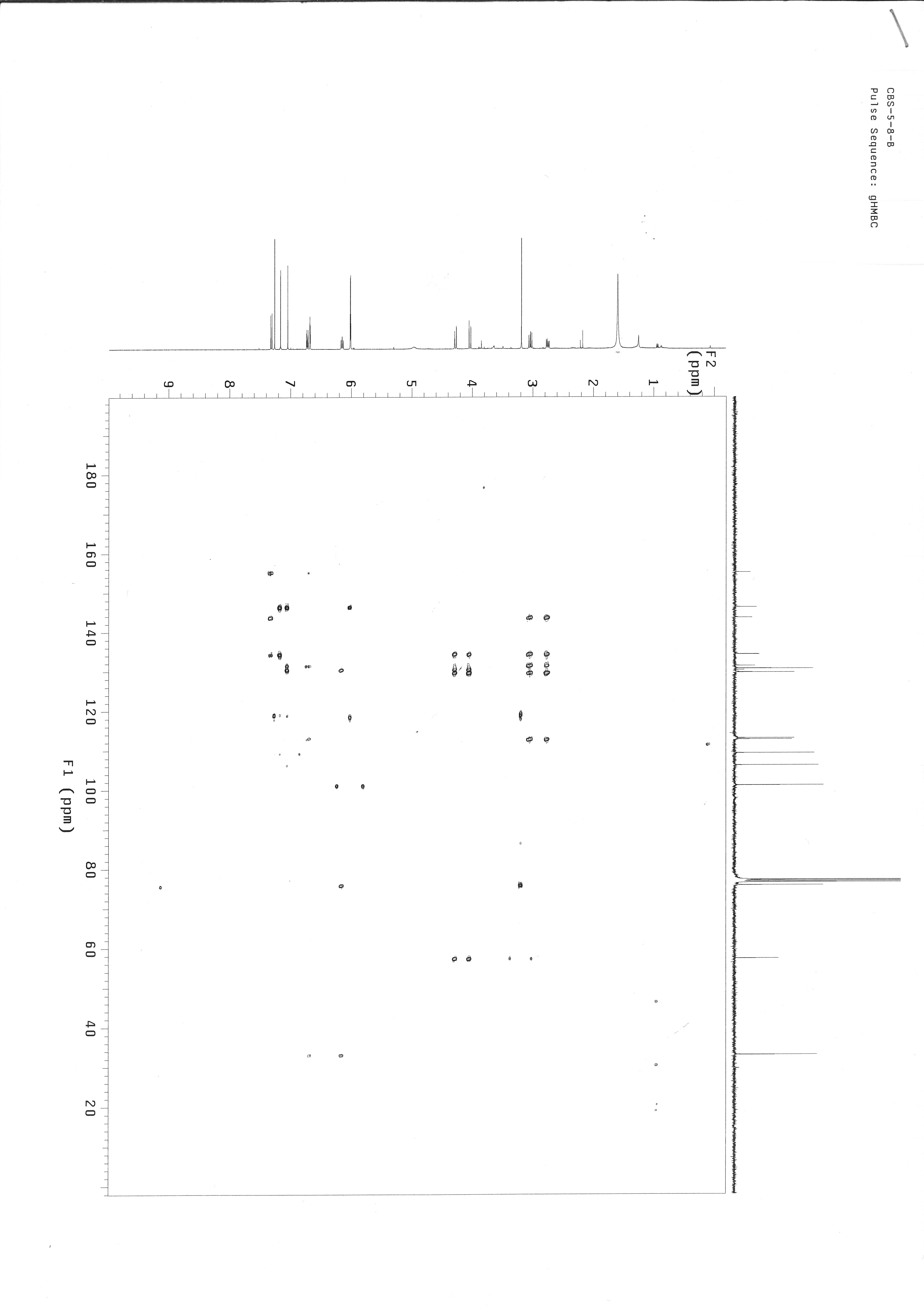


Figure A6. HMBC spectrum of **1.**


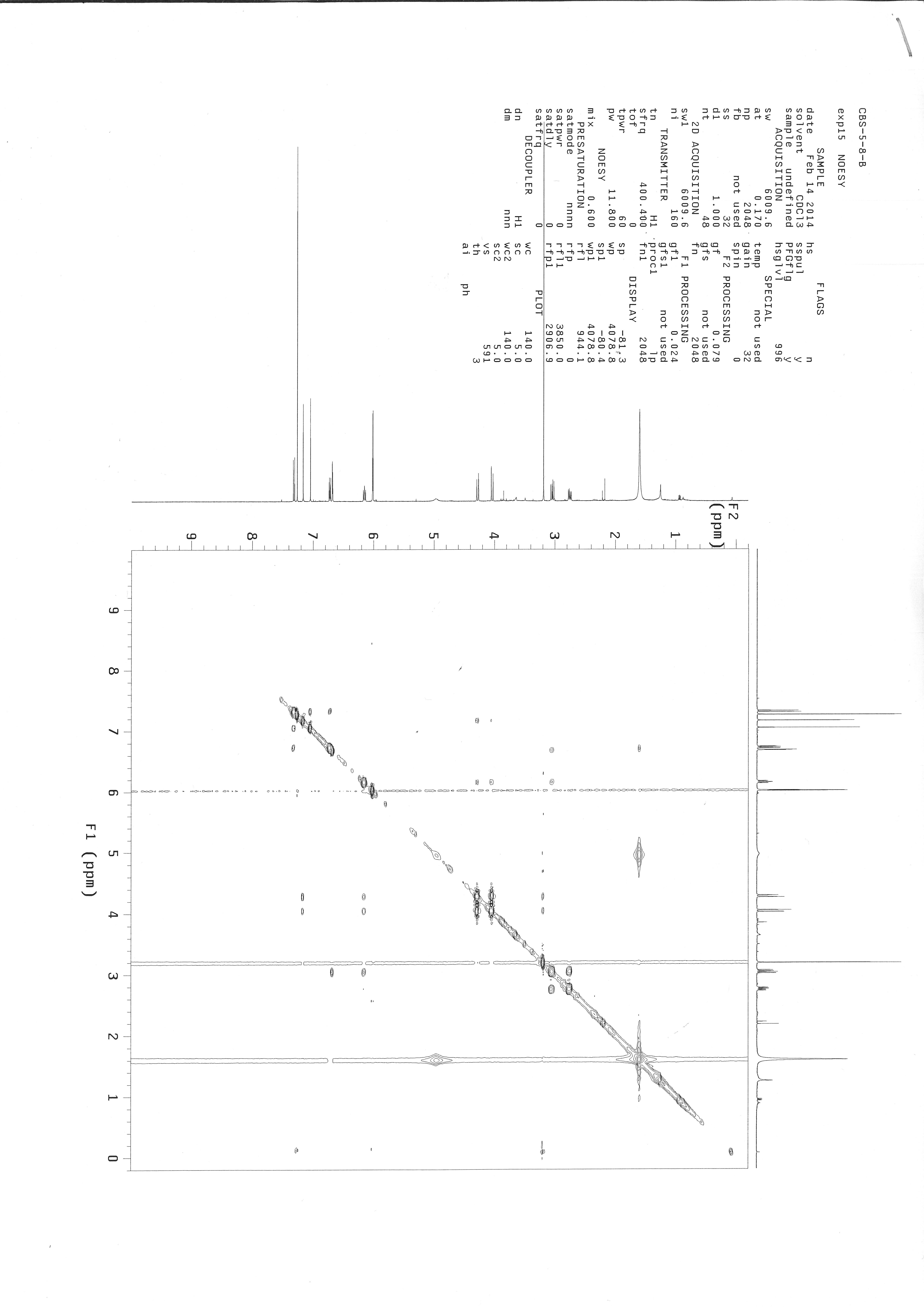


Figure A7. NOESY spectrum of **1.**
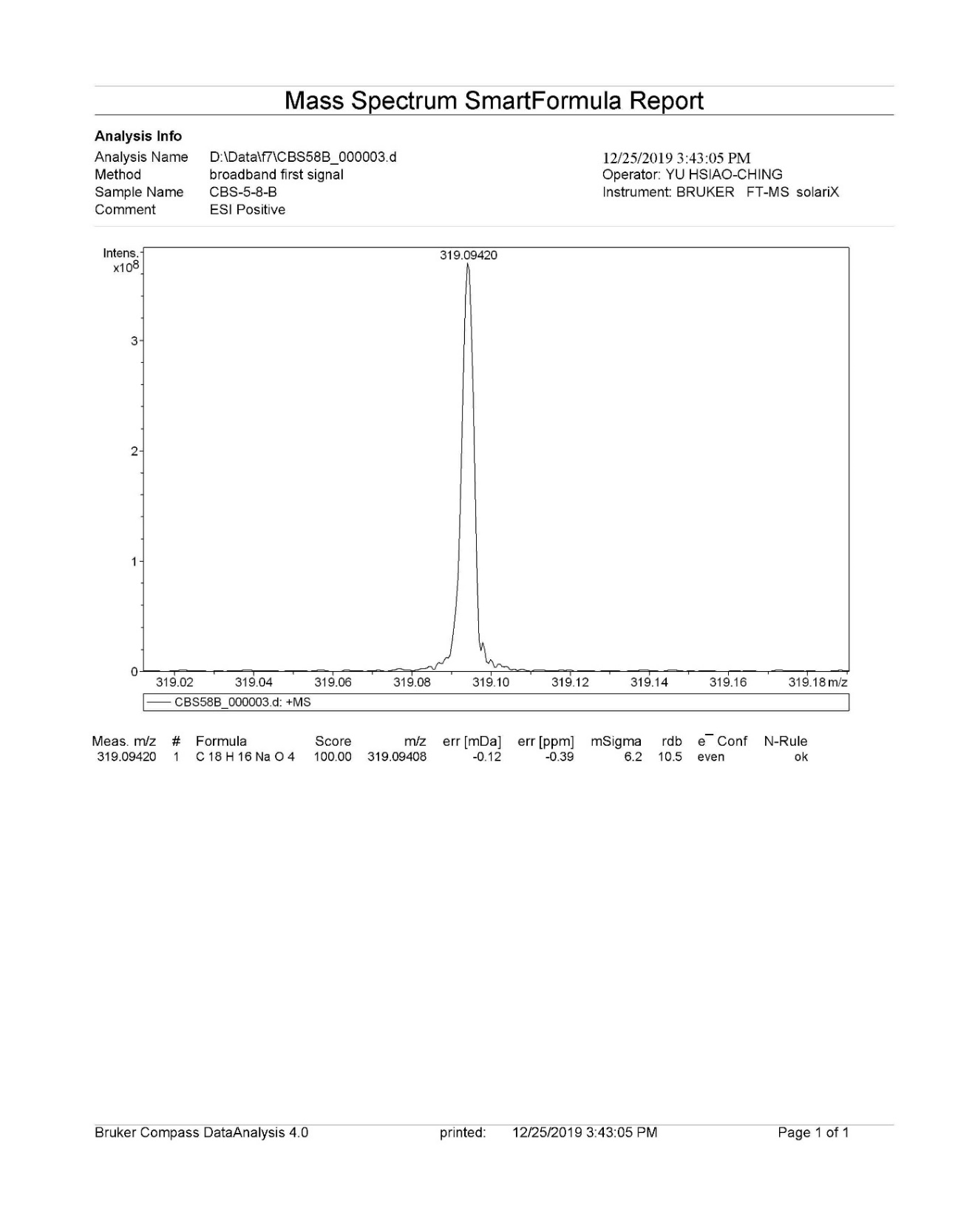


Figure A8. HRESIMS spectrum of **1.**


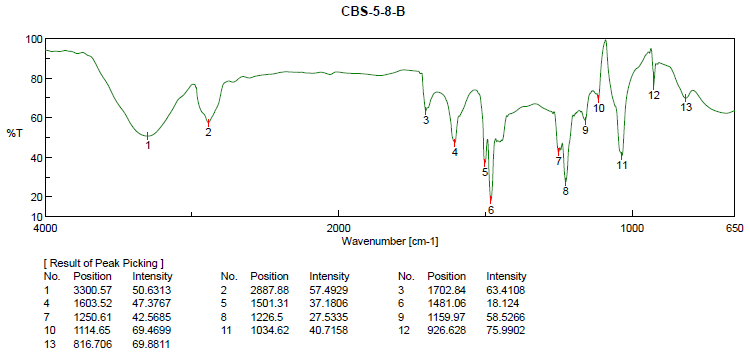


Figure A9. IR spectrum of **1.**


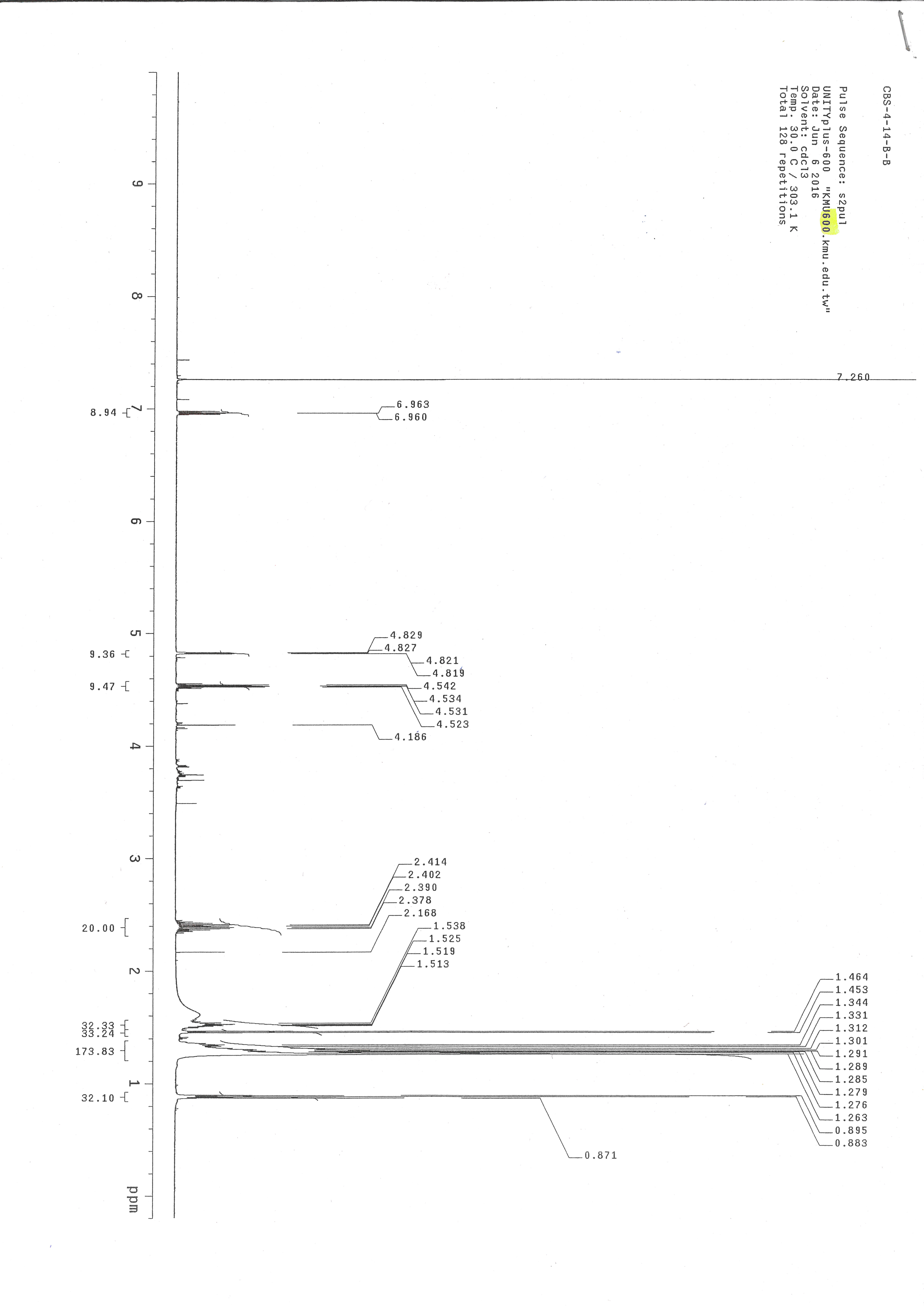


Figure A10. ^1^H NMR spectrum of **2** (600 MHz in CDCl_3_).


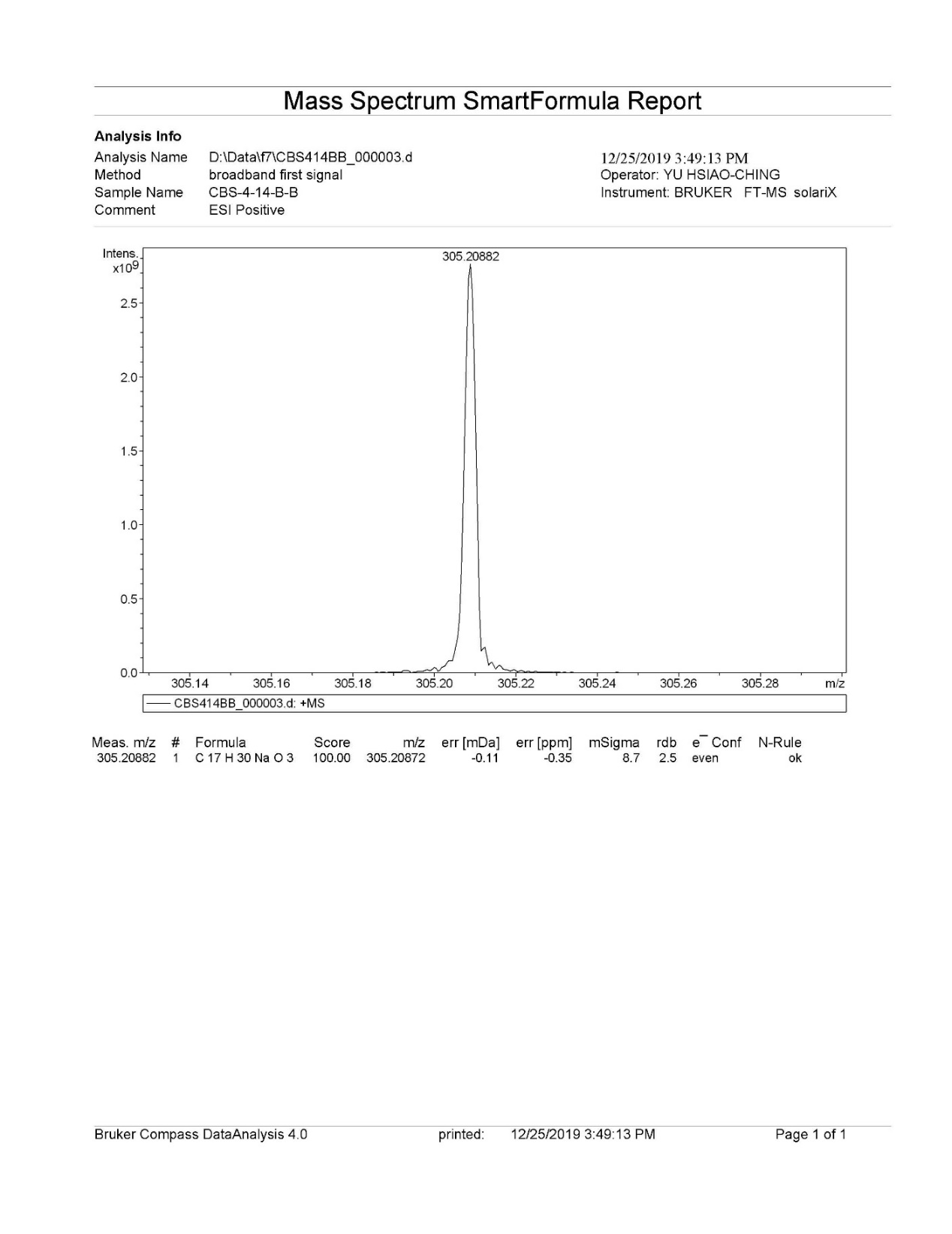


Figure A11. HRESIMS spectrum of **2.**


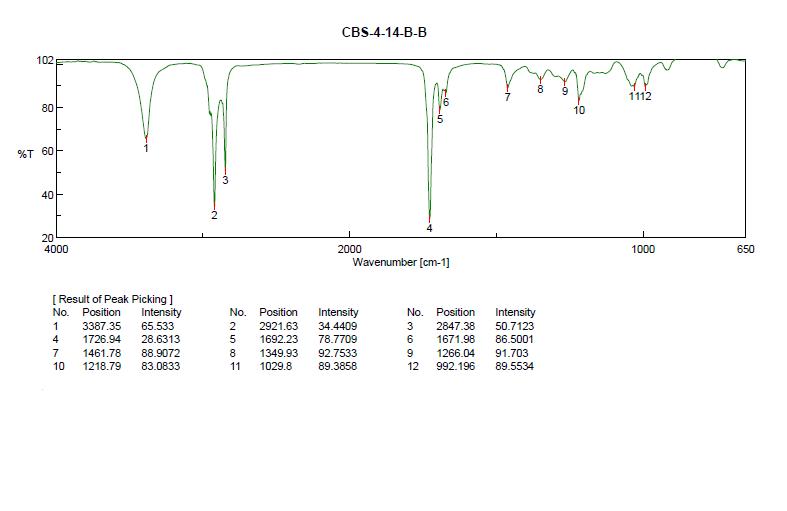


Figure A12. IR spectrum of **2.**

**Experimental data of known compounds**

**lincomolide A (3)**

C_17_H_30_O_3_ (282), Colorless oil, **[α]**^21^_D_ –36.9°(*c* 0.135; CHCl_3_), **UV** λ_max_ (MeOH) (log *ε*): 219 (4.05) nm, **IR** *υ*_max_ (ATR): 3485 (OH), 1731, 1677 (*α,β*-unsaturated-*γ*-lactone) cm^-1^, **^1^H NMR** (CDCl_3_, 200 MHz) *δ*: (ppm) 0.88 (3H, t, *J* = 6.4 Hz, H-17), 1.26 (l6H, s, H-9 – H-16), 1.45 (3H, d, *J* = 6.5 Hz, H-5), 1.59 (2H, m, H-8), 2.39 (2H, m, H-7), 4.54 (1H, qd, *J* = 6.5, 5.2 Hz, H-4), 4.81 (1H, br s, H-3), 6.96 (1H, td, *J* = 7.4, 2.0 Hz, H-6), **ESIMS** *m/z*: 283 [M+H] ^+^

**linderanolide B (4)**

C_19_H_32_O_3_ (308), colorless oil, **[α]^25^_D_** +1.32°(*c* 0.10; CHCl_3_), **UV** λ_max_ (MeOH) (log *ε*): 210 (4.09) nm, **IR** *υ*_max_ (ATR): 3416 (OH), 1676 (C=O) cm^-1^, **^1^H NMR** (CDCl_3_, 200 MHz) *δ*: (ppm) 0.88 (3H, t, *J =* 6.5 Hz, H-19), 1.25 (20H, s, H-9 – H-18), 1.48 (2H, m, H-8), 2.06 (1H, br s, OH-3), 2.77 (2H, br q, *J* = 7.8 Hz, H-7), 4.66 (1H, dd, *J =* 2.4, 1.9 Hz, H-5b), 4.89 (1H, dd, *J =* 2.4, 2.2 Hz, H-5a), 5.11 (1H, br s, H-3), 6.68 (1H, td, *J=* 7.8, 1.8 Hz, H-6), **ESIMS** *m/z*: 331 [M+Na]^+^

**isolinderanolide B (5)**

C_19_H_32_O_3_ (308), colorless oil, **[α]^21^_D_** +40.5° (*c* 0.10; CHCl_3_), **UV** λ_max_ (MeOH) (log *ε*): 217 (4.47), 260 sh (4.16) nm, **IR** *υ*_max_ (ATR): 3369 (OH), 1722 (*α,β*-unsaturated-*γ*-lactone) cm^-1^, **^1^H NMR** (CDCl_3_, 200 MHz) *δ*: (ppm) 0.87 (3H, t, *J* = 6.5 Hz, H-19), 1.25 (20H, br s, H-9 − H-18), 1.52 (2H, m, H-8), 2.45 (2H, m, H-7) 4.71 (1H, dd, *J =* 2.8, 1.3 Hz, H-5b), 4.95 (1H, dd, *J =* 2.8, 1.6 Hz, H-5a), 5.24 (1H, br s, H-3), 7.06 (1H, td, *J =* 7.9, 2.2 Hz, H-6), **ESIMS** *m/z*: 331 [M+Na] ^+^

**isophilippinolide A (6)**

C_23_H_40_O_3_ (364), colorless oil, **[α]**^22^_D_–13.5° (*c* 0.36; CHCl_3_), **UV** λ_max_ (MeOH) (log *ε*): 220 (4.59) nm, **IR** *υ*_max_ (ATR): 3402 (OH), 1741 (*α*,*β*-unsaturated *γ*-lactone) cm^-1^, **^1^H NMR** (CDCl_3_, 200 MHz) *δ*: (ppm) 0.88 (3H, t, *J* = 6.4 Hz, H-23), 1.25 (28H, br s, H-9 – H-22), 1.52 (2H, m, H-8), 2.09 (1H, br s, OH-3, D_2_O exchangeable), 2.47 (2H, m, H-7), 4.72 (dd, *J* = 2.8, 1.5 Hz, H-5b), 4.95 (dd, *J* = 2.8, 1.5 Hz, H-5a), 5.26 (1H, br s, H-3), 7.09 (1H, td, *J* = 7.9, 2.2 Hz, H-6), **ESIMS** *m/z*: 365 [M+H] ^+^

**secosubamolide (7)**

****C_20_H_36_O_4_ (340), pale yellowish oil, **[α]^22^_D_** +69.0° (*c* 0.28; CHCl_3_), **UV** λ_max_ (MeOH) (log *ε*): 213 (3.71) nm, **IR** *υ*_max_ (ATR): 3435 (OH), 1740 (ester), 1720 (ketone) cm^-1^, **^1^H NMR** (CDCl_3_, 200 MHz) *δ*: (ppm) 0.88 (3H, t, *J* = 6.5 Hz, H-16), 1.25 (20H, br s, H-6 – H-15), 1.51 (2H, m, H-5), 2.15 (3H, s, H-3'), 2.35 (2H, q, *J* = 7.6 Hz, H-4), 3.73 (3H, s, OCH_3_-1), 4.01 (1H, d, *J* = 5.0 Hz, OH-1', D_2_O exchangeable), 4.90 (1H, d, *J* = 5.0 Hz, H-1'), 7.08 (1H, t, *J* = 7.6 Hz, H-3), **ESIMS** *m/z*: 363 [M+Na]^+^

**reticuol (8)**

C_17_H_14_O_4_ (282), white amorphous solid, **UV** λ_max_ (MeOH) (log *ε*): 210 (3.92), 242 (3.98), 281 (3.50) nm, **UV** λ_max_ (MeOH+KOH) (log *ε*): 214 (4.53), 251 (3.86), 299 (3.68) nm, **IR** *υ*_max_ (ATR): 3318 (OH), 1605, 1487 (aromatic ring), 1039, 922 (OCH_2_O) cm^-1^, **^1^H NMR** (CDCl_3_, 200 MHz) *δ*: (ppm) 2.74 (1H, dd, *J* = 12.5, 6.6 Hz, H-5b), 3.03 (1H, dd, *J* = 12.5, 7.6 Hz, H-5a), 4.33 (1H, d, *J* = 12.7 Hz, H-12b), 4.50 (1H, d, *J* = 12.7 Hz, H-12a), 6.03 (2H, s, H-13), 6.16 (1H, dd, *J* = 7.6, 6.6 Hz, H-6), 6.72 (1H, dd, *J* = 8.4, 2.4, H-2), 6.78 (1H, d, *J* = 2.4 Hz, H-4), 7.07 (1H, s, H-8), 7.13 (1H, s, H-11), 7.33 (1H, d, *J* = 8.4 Hz, H-1), **EIMS** (rel. int. %) *m/z*: 282 [M]^+^ (36), 264 (14), 251 (100), 221 (14), 193 (l2), 165 (30)

**burmanol (9)**

C_17_H_16_O_4_ (284), colorless oil, **UV** λ_max_ (MeOH) (log *ε*): 209 (3.71), 239 (3.64), 266 sh (3.20) nm, **IR** *υ*_max_ (ATR): 3395 (OH), 1668, 1596, 1512 (aromatic ring) cm^-1^, **^1^H NMR** (CDCl_3_, 200 MHz) *δ*: (ppm) 2.80 (1H, dd, *J* = 12.6, 6.6 Hz, H-5b), 3.14 (1H, dd, *J* = 12.6, 7.7 Hz, H-5a), 3.99 (3H, s, H-13), 4.36 (1H, d, *J* = 13.4 Hz, H-12b), 4.53 (1H, d, *J* = 13.4 Hz, H-12a), 5.69 (2H, br s, OH-3, OH-9, D_2_O exchangeable), 6.19 (1H, dd, *J* = 7.7, 6.6 Hz, H-6), 7.12 (1H, s, H-8), 7.22 (1H, s, H-11), 7.25 (1H, d, *J* = 2.2, H-4), 7.30 (1H, dd, *J* = 9.0, 2.2 Hz, H-2), 7.51 (1H, d, *J* = 9.0 Hz, H-1), **ESIMS** *m/z*: 307 [M+Na] ^+^

**(–)-5,7-dimethoxy-3′,4′-methylenedioxy-flavan-3-ol (10)**

C_18_H_18_O_6_ (330), colorless needles (MeOH), **M.p.:** 146 − 147ºC, **[α]**^22^_D_– 25.2° (*c* 0.06; CHCl_3_), **UV** λ_max_ (MeOH) (log *ε*): 207 (3.96), 230 sh (3.40), 284 (2.92) nm, **IR** *υ*_max_ (ATR): 3405 (OH), 1620, 1592, 1493 (aromatic ring), 1038, 921 (OCH_2_O) cm^-1^, **^1^H NMR** (CDCl_3_, 400 MHz) *δ*: (ppm) 2.91 (2H, m, H-4), 3.77 (3H, s, OCH_3_-7), 3.80 (3H, s, OCH_3_-5), 4.26 (1H, br s, H-3), 4.93 (1H, br s, H-2), 5.98 (2H, s, H-7'), 6.12 (1H, d, *J =* 2.2 Hz, H-6), 6.18 (1H, d, *J =* 2.2 Hz, H-8), 6.85 (1H, d, *J =* 8.0 Hz, H-5'), 6.97 (1H, dd, *J* = 8.0, 1.4, H-6'), 7.05 (1H, d, *J* = 1.4 Hz, H-2'), **EIMS** (rel. int. %) *m/z* : 330 [M]^+^ (28), 167 (100), 135 (28), 109 (12)

**taxifolin (11)**

****C_15_H_12_O_7_ (304), yellowish amorphous solid, **[α]**^21^_D_ +60.0° (*c* 0.15; MeOH), **UV** λ_max_ (MeOH) (log *ε*): 206 (4.22), 229 sh (3.98), 289 (3.93) nm, **UV** λ_max_ (MeOH +KOH) (log *ε*): 207 (4.25), 325 (3.64) nm, **IR** *υ*_max_ (ATR): 3370 (OH), 1642 (C=O), 1518, 1464 (aromatic ring) cm^-1^, **^1^H NMR** (CD_3_OD, 200 MHz) *δ*: (ppm) 4.50 (1H, d, *J* = 11.6 Hz, H-3), 4.91 (1H, d, *J* = 11.6 Hz, H-2), 5.88 (1H, d, *J* = 2.0 Hz, H-6), 5.92 (1H, d, *J* = 2.0 Hz, H-8), 6.80 (1H, d, *J* = 8.2 Hz, H-5'), 6.85 (1H, dd, *J* = 8.2, 1.9 Hz, H-6'), 6.96 (1H, d, *J* = 1.9 Hz, H-2'), **^13^C NMR** (CD_3_OD, 50 MHz) *δ*: (ppm) 73.6 (C-3), 85.1 (C-2), 96.3 (C-8), 97.3 (C-6), 101.8 (C-10), 115.9 (C-5'), 116.1 (C-2'), 120.9 (C-6'), 129.8 (C-1'), 146.3 (C-3'), 147.1 (C-4'), 164.5 (C-9), 165.3 (C-5), 168.7 (C-7), 198.4(C-4), **ESIMS** *m/z*: 305 [M+H] ^+^

**(–)-yangambin (12)**

****C_24_H_30_O_8_ (446), colorless oil, **[α]^22^_D_** –35.8° (*c* 0.03; CHCl_3_), **UV** λ_max_ (MeOH) (log *ε*): 210 (4.67), 234 sh (4.15), 272 (3.71) nm, **IR** *υ*_max_ (ATR): 1590, 1508, 1460 (aromatic ring), 1125 (C-O) cm^-1^, **^1^H NMR** (CDCl_3_, 200 MHz) *δ*: (ppm) 3.11 (2H, m, H-1, H-5), 3.84 (6H, s, OCH_3_-4′, OCH_3_-4″), 3.88 (l2H, s, OCH_3_-3′, OCH_3_-5′, OCH_3_-3″, OCH_3_-5″), 3.94 (2H, dd, *J* = 9.2, 3.9 Hz, H-4b, H-8b), 4.31 (2H, dd, *J* = 9.2, 6.9 Hz, H-4a, H-8a), 4.75 (2H, d, *J* = 4.4 Hz, H-2, H-6), 6.57 (4H, s, H-2′, H-6′, H-2″, H-6″), **EIMS** (rel. int. %) *m/z*: 446 [M]^+^ (88), 265 (13), 235 (18), 224 (32), 207 (76), 181(100), 176 (32), 151 (26)

**(–)-pinoresinol (13)**

****C_20_H_22_O_6_ (358), white amorphous powder, **[α]^22^_D_**–12.0° (*c* 0.35; CHCl_3_), **UV** λ_max_ (MeOH) (log *ε*): 205 (4.60), 231 (4.03), 280 (3.66) nm, **UV** λ_max_ (MeOH+KOH) (log *ε*): 211 (4.53), 252 (3.92), 292 (3.48) nm, **IR** *υ*_max_ (ATR): 3368 (OH), 1608, 1513, 1432, (aromatic ring) cm^-1^, **^1^H NMR** (CD_3_OD, 200 MHz) *δ*: (ppm) 3.14 (2H, m, H-1, H-5), 3.82 (2H, m, H-4b, H-8b), 3.86 (6H, s, OCH_3_-3′, OCH_3_-3″), 4.23 (2H, dd, *J* = 8.9, 6.9 Hz, H-4a, H-8a), 4.71 (2H, d, *J* = 4.6 Hz, H-2, H-6), 6.76 (2H, d, *J* = 8.2 Hz, H-5′, H-5″), 6.76 (2H, dd, *J* = 8.2, 1.6 Hz, H-6′, H-6″), 6.95 (2H, d, *J* = 1.6 Hz, H-2′, H-2″), **EIMS** (rel. int. %) *m/z*: 358 [M]^+^ (24), 207 (10), 163 (l6), 152 (20), 151 (100), 137 (38), 131(26)

**(+)-monomethylpinoresinol (14)**

C_21_H_24_O_6_ (372), pale yellowish amorphous solid, [α]^22^_D_ +45.3° (*c* 0.02; CHCl_3_), UV λ_max_ (MeOH) (log *ε*): 206 (4.22), 229 (3.79), 279 (3.34) nm, **UV** λ_max_ (MeOH+KOH) (log *ε*): 209 (4.44), 251 sh (3.45), 301 sh (2.92) nm, **IR** *υ*_max_ (ATR): 3397 (OH), 1596, 1517 (aromatic ring) cm^-1^, **^1^H NMR** (CDCl_3_, 200 MHz) *δ*: (ppm) 3.11 (2H, m, H-1, H-5), 3.88 (3H, s, OCH_3_-3"), 3.90 (3H, s, OCH_3_-4'), 3.91 (3H, s, OCH_3_-3'), 3.95 (2H, m, overlapped with OMe signal, H-4b, H-8b), 4.25 (2H, m, H-4a, H-8a), 4.74 (2H, m, H-2, H-6), 5.59 (1H, s, OH-4", D_2_O exchangeable), 6.85 – 6.90 (6H, m, H-2′, H-5′, H-6′, H-2″, H-5″, H-6″), **ESIMS** *m/z*: 373 [M+H] ^+^

**(+)-syringaresinol (15)**

C_22_H_26_O_8_ (418), colorless needles (MeOH), **M.p.**: 166 − 168ºC, **[α]^22^_D_** +25.3° (*c* 0.10; CHCl_3_), **UV** λ_max_ (MeOH) (log *ε*): 211 (4.53), 239 (3.97), 271 (3.25) nm, **UV** λ_max_ (MeOH +KOH) (log *ε*): 215 (4.69), 259 (4.21) nm, **IR** *υ*_max_ (ATR): 3395 (OH), 1610, 1516, 1457 (aromatic ring) cm^-1^, **^1^H NMR** (CD_3_OD, 200 MHz) *δ*: (ppm) 3.15 (2H, m, H-1, 5), 3.85 (12H, s, OCH_3_-3', 3'', 5', 5''), 3.87 − 3.93 (2H, m, H-4b, H-8b), 4.27 (2H, dd, *J* = 9.1, 6.9 Hz, H-4a, H-8a), 4.72 (2H, d, *J* = 4.4 Hz, H-2, H-6), 6.66 (4H, s, H-2', 2'', 6', 6''), **ESIMS** *m/z*: 419 [M+H] ^+^

***erythro*-guaiacylglycerol-*β*-*O*-4'-(5')-methoxylariciresinol (16)**

******C_31_H_38_O_11_(586), colorless oil, **[α]**^22^_D_ +16.7°(*c* 0.07; MeOH), **UV** λ_max_ (MeOH) (log *ε*): 210 (4.47), 229 (4.22) sh, 279 (3.78), **UV** λ_max_ (MeOH+KOH) (log *ε*): 215 (4.62), 244 (4.17), 292 (3.76) nm, **IR** *υ*_max_ (ATR): 3351 (OH), 1593, 1515, 1461 (aromatic ring) cm^-1^, **^1^H NMR** (acetone-*d*_6_, 600 MHz) *δ*: (ppm) 2.34 (1H, m, H-8′), 2.55 (1H, dd, *J* = 13.7, 10.5 Hz, H-7b), 2.71 (1H, m, H-8), 2.93 (1H, dd, *J* = 13.7, 5.4 Hz, H-7a), 3.37 (1H, ddd, *J* = 7.2, 5.4, 3.6 Hz, OH-9"), 3.43 (1H, ddd, *J* = 10.5, 6.9, 3.3 Hz, H-9"b), 3.59 (1H, m, OH-9′), 3.70 (1H, dd, *J* = 8.1, 4.2 Hz, H-9b), 3.74 (1H, ddd, *J* = 10.5, 7.2, 5.1 Hz, H-9′b), 3.82 (3H, s, OCH_3_-3"), 3.84 (3H, s, OCH_3_-3), 3.84 (6H, s, OCH_3_-3′, OCH_3_-5′ ), 3.84 (1H, m, overlapped OCH_3_, H-9"a), 3.90 (1H, m, H-9′a), 4.00 (1H, dd, *J* = 8.1, 6.6 Hz, H-9a), 4.16 (1H, ddd, *J* = 8.4, 6.3, 3.6 Hz, H-8"), 4.34 (1H, d, *J* = 4.2 Hz, OH-7"), 4.89 (1H, d, *J* = 6.0 Hz, H-7′), 4.98 (1H, dd, *J* = 8.4, 4.2 Hz, H-7"), 6.66 (1H, dd, *J* = 8.1, 1.8 Hz, H-6), 6.73 (2H, br s, H-2′, H-6′), 6.74 (1H, d, *J* = 8.1 Hz, H-5), 6.77 (1H, d, *J* = 8.1 Hz, H-5"), 6.83 (1H, dd, *J* = 8.1, 1.8 Hz, H-6"), 6.83 (1H, d, *J* = 1.8 Hz, H-2), 7.05 (1H, d, *J* = 1.8 Hz, H-2"), 7.28 (1H, br s, OH-4), 7.35 (1H, br s, OH-4"), **^13^C NMR** (acetone-*d*_6_, 150 MHz) *δ*: (ppm) 34.1 (C-7), 44.1 (C-8), 54.5 (C-8'), 57.0 (OCH_3_-3, OCH_3_-3"), 57.2 (OCH_3_-3', OCH_3_-5'), 61.3 (C-9'), 61.7 (C-9"), 74.0 (C-9), 74.1 (C-7"), 84.1 (C-7'), 88.6 (C-8"), 104.6 (C-2', C-5'), 111.7 (C-2"), 113.7 (C-2), 115.9 (C-5"), 116.4 (C-5), 120.7 (C-6"), 122.5 (C-6), 134.0 (C-1), 134.5 (C-1"), 136.1 (C-4'), 142.3 (C-1'), 146.4 (C-4), 147.2 (C-4"), 148.7 (C-3"), 149.0 (C-3), 154.7 (C-3', C-5'), **ESIMS** *m/z*: 609 [M+Na]^+^

**caryolane-1,9*β*-diol (17)**

****C_15_H_26_O_2_ (238), colorless oil, **[α]**^20^_D_ –36.6° (*c* 0.09; CHCl_3_), **IR** *υ*_max_ (ATR): 3405 (OH) cm^-1^, **^1^H NMR** (CDCl_3_, 600 MHz) *δ*: (ppm) 0.93 (3H, s, H-15), 1.00 (3H, s, H-13), 1.02 (3H, s, H-14), 1.15 (1H, m, H-7b), 1.38 (1H, m, H-6b), 1.42 (1H, m, H-7a), 1.42 (1H, d, *J =* 13.2 Hz, H-12b), 1.48 (1H, d, *J =* 13.2 Hz, H-12a), 1.49 (1H, dd, *J =* 12.6, 10.2 Hz, H-3b), 1.50 (1H, m, H-11b), 1.51 (1H, m, H-6a), 1.52 (1H, t, *J =* 12.6 Hz, H-3a), 1.65 (1H, td, *J =* 12.6, 6.5 Hz, H-11a), 1.77 (1H, ddt, *J =* 15.3, 6.5, 3.5 Hz, H-10b), 1.89 (1H, ddd, *J* = 12.5, 8.7, 6.6 Hz, H-5), 2.04 (1H, m, H-10a), 2.22 (1H, ddd, *J =* 12.5, 10.2, 8.4 Hz, H-2), 3.44 (1H, t, *J =* 3.5 Hz, H-9), **^13^C NMR** (CDCl_3_, 150 MHz) *δ*: (ppm) 20.5 (C-6), 20.8 (C-13), 26.6 (C-15), 28.3 (C-10), 30.5 (C-14), 33.5 (C-11), 34.1 (C-3), 35.2 (C-4), 35.5 (C-7), 38.2 (C-2), 39.4 (C-8), 42.6 (C-12), 44.0 (C-5), 70.8 (C-1), 72.3 (C-9), **ESIMS** *m/z*: 261 [M+Na]^+^

***β*-sitosterol (18)**

******C_29_H_50_O (414), colorless needles (MeOH), **M.p.**: 129 − 131ºC, **[α]**^21^_D_–20.5° (*c* 0.05; CHCl_3_), **IR** *υ*_max_ (ATR): 3408 (OH) cm^-1^, **^1^H NMR** (CDCl_3_, 200 MHz) *δ*: (ppm) 0.68 (3H, s, H-18), 0.81 (3H, d, *J* = 6.6 Hz, H-26), 0.83 (3H, d, *J* = 6.6 Hz, H-27), 0.84 (3H, t, *J* = 7.7 Hz, H-29), 0.92 (3H, d, *J* = 6.4 Hz, H-21), 1.00 (3H, s, H-19), 3.52 (1H, m, H-3), 5.35 (1H, br d, *J* = 5.2 Hz, H-6)

**cinnamtannin B1 (19)**

****C_45_H_36_O_18_ (864), brownish amorphous solid, **[α]**^21^_D_ +127.4° (*c* 0.25; MeOH), **UV** λ_max_ (MeOH) (log *ε*): 233 (5.25), 280 (4.97) nm, **UV** λ_max_ (MeOH+KOH) (log *ε*): 243 (5.32), 290 sh (5.10) nm, **IR** *υ*_max_ (ATR): 3384 (OH), 1615, 1522, 1450 (aromatic ring) cm^-1^, **^1^H NMR** (CD_3_OD, 400 MHz) *δ*: (ppm) 2.84 (2H, m, H-t-4), 3.29 (1H, d, *J* = 3.6 Hz, H-u-3), 3.86 (1H, br t, *J* = 2.8 Hz, H-t-3), 4.13 (1H, br d, *J* = 2.0 Hz, H-m-3), 4.15 (1H, d, *J* = 3.6 Hz, H-u-4), 4.39 (1H, br s, H-t-2), 4.56 (1H, br s, H-m-4), 5.71 (1H, br s, H-m-2), 5.81 (1H, s, H-m-6), 5.97 (1H, d, *J* = 2.2 Hz, H-u-6), 6.02 (1H, d, *J* = 2.2 Hz, H-u-8), 6.10 (1H, s, H-t-6), 6.73 (1H, dd, *J* = 8.2, 1.8 Hz, H-t-6'), 6.76 (1H, d, *J* = 8.2 Hz, H-t-5'), 6.82 (1H, d, *J* = 8.4 Hz, H-m-5'), 6.83 (1H, d, *J* = 1.8 Hz, H-t-2'), 6.84 (1H, d, *J* = 8.4 Hz, H-u-5'), 6.86 (1H, dd, *J* = 8.4, 2.0 Hz, H-u-6'), 7.04 (1H, d, *J* = 2.0 Hz, H-u-2'), 7.19 (1H, dd, *J* = 8.4, 2.0 Hz, H-m-6'), 7.32 (1H, d, *J* = 2.0 Hz, H-m-2'), **^13^C NMR** (CD_3_OD, 100 MHz) *δ*: (ppm) 28.86 (C-u-4), 29.85 (C-t-4), 38.27 (C-m-4), 67.17 (C-t-3), 67.52 (C-u-3), 72.57 (C-m-3), 78.86 (C-m-2), 80.28 (C-t-2), 96.05 (C-m-6), 96.44(C-t-6), 96.55(C-u-8), 98.29 (C-u-6), 99.94 (C-u-2), 100.03 (C-t-10), 104.94 (C-u-10), 106.41 (C-m-8), 106.71 (C-m-10), 108.84 (C-t-8), 115.23 (C-t-2'), 115.47 (C-u-2'), 115.74 (C-m-5'), 115.99 (C-t-5'), 116.13 (C-u-5'), 116.72 (C-m-2'), 119.42 (C-t-6'), 119.86 (C-u-6'), 121.35 (C-m-6'), 131.77 (C-m-1'), 132.45 (C-u-1'), 133.17 (C-t-1'), 145.32 (C-t-3'), 145.46 (C-u-3'), 145.75 (C-t-4'), 145.88 (C-m-3'), 146.27 (C-m-4'), 146.60 (C-u-4'), 151.08 (C-m-7), 151.79 (C-m-9), 154.15 (C-u-9), 155.55 (C-t-7), 155.76 (C-m-5), 155.80 (C-t-9), 156.02 (C-t-5), 156.76 (C-u-5), 157.83 (C-u-7), **ESIMS** *m/z*: 865 [M+H] ^+^
